# Spatial and dosimetric evaluation of residual distortions of prostate and seminal vesicle bed after image‐guided definitive and postoperative radiotherapy of prostate cancer with endorectal balloon

**DOI:** 10.1002/acm2.13138

**Published:** 2020-12-30

**Authors:** Sabine Levegrün, Christoph Pöttgen, Konstantinos Xydis, Maja Guberina, Jehad Abu Jawad, Martin Stuschke

**Affiliations:** ^1^ Department of Radiotherapy University Hospital Essen Essen Germany

**Keywords:** accumulated dose distributions, image‐guided radiotherapy, prostate cancer, residual distortions, time series

## Abstract

**Purpose:**

To quantify daily residual deviations from the planned geometry after image‐guided prostate radiotherapy with endorectal balloon and to evaluate their effect on the delivered dose distribution.

**Methods:**

Daily kV‐CBCT imaging was used for online setup‐correction in six degrees of freedom (6‐dof) for 24 patients receiving definitive (12 RT_def_ patients) or postoperative (12 RT_postop_ patients) radiotherapy with endorectal balloon (overall 739 CBCTs). Residual deviations were evaluated using several spatial and dosimetric variables, including: (a) posterior Hausdorff distance HD^post^ (=maximum distance between planned and daily CTV contour), (b) point P_worst_ with largest HD^post^ over all fractions, (c) equivalent uniform dose using a cell survival model (EUD_SF_) and the generalized EUD concept (gEUD_a_ with parameter a = −7 and a = −20). EUD values were determined for planned (${\text{EUD}}_{\text{SF}}^{\text{plan}}$), daily (${\text{EUD}}_{\text{SF}}^{\text{ind}}$), and delivered dose distributions (${\text{EUD}}_{\text{SF}}^{\text{accum}}$) for plans with 6 mm (=clinical plans) and 2 mm CTV‐to‐PTV margin. Time series analyses of interfractional spatial and dosimetric deviations were conducted.

**Results:**

Large HD^post^ values ≥ 12.5 mm (≥15 mm) were observed in 20/739 (5/739) fractions distributed across 7 (3) patients. Points P_worst_ were predominantly located at the posterior CTV boundary in the seminal vesicle region (16/24 patients, 6/7 patients with HD^post^ ≥ 12.5 mm). Time series analyses revealed a stationary white noise characteristic of HD^post^ and relative dose at P_worst_. The EUD_SF_ difference between planned and accumulated dose distributions was < 5.4% for all 6‐mm plans. Evaluating 2‐mm plans, EUD_SF_ deteriorated by < 10% (<5%) in 75% (58.5%) of the patients. ${\text{EUD}}_{\text{SF}}^{\text{accum}}$ was well described by the median value of the ${\text{EUD}}_{\text{SF}}^{\text{ind}}$ distribution. PTV margin calculation at P_worst_ yielded 8.8 mm.

**Conclusions:**

Accumulated dose distributions in prostate radiotherapy with endorectal balloon are forgiving of considerable residual distortions after 6‐dof patient setup if they are observed in a minority of fractions and the median value of ${\text{EUD}}_{\text{SF}}^{\text{ind}}$ determined per fraction stays within 95% of prescribed dose. Common PTV margin calculations are overly conservative because after online correction of translational and rotational errors only residual deformations need to be included. These results provide guidelines regarding online navigation, margin optimization, and treatment adaptation strategies.

## 1 INTRODUCTION

Advanced radiotherapy hardware and software solutions enable daily image guidance for online patient position correction and, in addition, allow the evaluation of residual deviations by means of deformable image registrations and dose summation tools with the purpose of comparing planned and actually delivered dose distributions and optimizing PTV margins. Prostate cancer is a site particularly prone to interfraction motion and distortions, and the choice of posterior margins critically affects rectal toxicity.

The application of an endorectal balloon in prostate radiotherapy has been associated with a number of advantages. Endorectal balloons have been shown to decrease the rectal and anal wall volumes being irradiated to intermediate and high doses by pushing the lateral and posterior rectal wall out of the high‐dose region.^1^, ^2^, ^3^, ^4^ This was associated with a lower incidence of late rectal toxicity.^3^, ^4^ In addition, an endorectal balloon reduced intrafraction prostate motion.^5^, ^6^, ^7^ On the other hand, the use of endorectal balloons may affect the geometrical accuracy of treatment delivery. Studies using balloon‐type endorectal MRI coils demonstrated that endorectal balloons can cause deformations of the prostate.^8^, ^9^ Moreover, analyses of the interfraction variability in endorectal balloon placement relative to the prostate or bony landmarks reported substantial deviations in shape and position from the nominal geometry.^10^, ^11^, ^12^ The intra‐ and interindividual dosimetric consequences of such residual deformations and repositioning inaccuracies on the delivered dose distributions in prostate radiotherapy with endorectal balloon are subject of this paper.

We have analyzed a cohort of prostate cancer patients who received definitive or postoperative image‐guided radiotherapy (IGRT) with endorectal balloon. Image guidance was based on daily kV cone beam CTs (CBCT) acquired before treatment and subsequent online position correction with a six degrees of freedom (6‐dof) couch using a standardized matching region of interest. The aim was to determine the resulting geometrical accuracy of the CTV position in each treatment fraction and to quantify dosimetric consequences of residual deviations from the planned geometry on the delivered dose distribution using deformable image registrations and dose accumulation. In addition, we evaluated the development of residual interfractional distortions as function of time over the course of treatment. Finally, we assessed the size of the posterior CTV‐to‐PTV margin required to assure an adequate dose coverage of the prostate according to both spatial and dosimetric criteria.

## 2 METHODS

### 2.A Patient dataset

#### 2.A.1 Patient characteristics

The dataset included 24 prostate cancer patients who received definitive radiotherapy (RT_def_, 12 consecutive patients, denoted by patient numbers 1–12) or were treated after prostatectomy for PSA recurrence or with adjuvant radiotherapy in case of positive resection margins (RT_postop_, 12 consecutive patients, denoted by patient numbers 101–112). This retrospective study was approved by the ethics committee of our institution.

#### 2.A.2 Treatment planning

Computer tomography (CT) imaging for treatment planning was acquired in supine position with emptied rectum and half‐filled urinary bladder (approx. 200 ml). All patients were imaged and treated with an endorectal balloon fabricated in‐house in two sizes (small: 10 cm length, 3.6 cm diameter, 23 patients; large: 13.5 cm length, 4.6 cm diameter, 1 patient). Prior to CT imaging and each fraction, the endorectal balloon was covered with anesthesia gel, manually inserted and inflated to a prescribed fill volume of 75 ml (small size) or 125 ml air (large size) using a 50 ml syringe. The endorectal balloon was then gently retracted towards the anal canal.

Treatment planning was performed in Eclipse (V15.5, Varian Medical Systems, Palo Alto, CA, USA). All patients were treated with 6 or 8 MV photons using a volumetric modulated arc therapy technique (RapidArc, Varian Medical Systems, Palo Alto, CA, USA) with two counter‐rotating arcs.

RT_def_ patients received a total dose of 78 Gy delivered in 39 fractions (5 × 2 Gy per week) to the CTV containing the prostate and base of the seminal vesicles. RT_postop_ patients were treated to 68 Gy delivered in 34 fractions of 2 Gy or to 68.4 Gy in 38 fractions of 1.8 Gy. When an initial CTV1 was treated which included the seminal vesicles or seminal vesicle base followed by a second series excluding the seminal vesicles in CTV2, only CTV1 was analyzed. The PTV was calculated as CTV expansion by 6 mm posteriorly and 8 mm in anterior and lateral direction.

#### 2.A.3 Treatment delivery and daily image guidance

Treatments were delivered at a Novalis TrueBeam linac (Varian Medical Systems, Palo Alto, CA, USA; BrainLAB AG, Feldkirchen, Germany) equipped with a 6‐dof Perfect Pitch couch top. Prior to each fraction, a low‐dose CBCT was recorded with acquisition parameters optimized to balance image quality and dose with preponderance of high contrast structures, i.e. bones and the air‐filled endorectal balloon (X‐ray tube current: 25 mA, voltage: 125 kV, exposure: 335 mAs, exposure time: 13.425 s, full arc, CTDI value: 4.5 mGy, 2 mm slice thickness, 512 × 512 image matrix). A rigid 6‐dof registration between planning CT and CBCT was performed automatically using a rectangular region of interest comprising the anterior half of the endorectal balloon posteriorly, the symphysis anteriorly, the periprostatic tissue and the obturator internus muscle laterally, the seminal vesicles superiorly, and the penile bulb inferiorly (“online match”). The obtained 6‐dof correction vector was used for online adjustment of the patient position using the treatment couch.

Patients received on average 30.8 CBCTs during treatment (range: 23–37 CBCTs per patient). The 12 RT_def_ patients had 346 CBCTs (range: 23–33 CBCTs per patient). The 12 RT_postop_ patients had 393 CBCTs (range: 28–37 CBCTs). In total, 739 CBCTs were analyzed.

### 2.B Data analysis

To assess residual setup errors not correctable by rigid registration, deformable image registrations were performed offline between the planning CT (i.e., reference image) and each CBCT (i.e., target image). The deformable image registration software implemented in Eclipse uses a modified, accelerated demons algorithm.^13^, ^14^ Each deformable registration was based on the rigid registration of the online match. To assess the daily variation in patient anatomy, the resulting registration vector field was used after visual inspection to propagate the planned CTV contour CTV_plan_ from the planning CT to each of the n CBCTs performed per patient, yielding contours CTV_CBCTi_ (i = 1,…,n). The CTV_CBCTi_ contours were then rigidly copied back to the planning CT using the online match for further analysis.

#### 2.B.1 Spatial evaluations

For every patient, the union CTV_acc_ of CTV_plan_ and all CTV_CBCTi_ was calculated. To evaluate how far the daily CTV_CBCTi_ contours projected beyond CTV_plan_, a series of evaluation PTVs was generated with isotropic margins of 2 to ≥20 mm (step width 1 mm) around CTV_plan_ (PTV^xmm^), and shells of 1 mm width were derived. To geometrically assess the dislocation of the daily CTV contour, the absolute volume of each CTV_CBCTi_ outside of PTV^xmm^ (x = 2,…,≥20 mm) was determined. From these data, we derived for all patients and every fraction the Hausdorff distance HD^iso^ (superscript iso: isotropic), i.e. the largest of all the distances from a point on CTV_plan_ to the closest point on CTV_CBCTi_, as a measure of the maximum shift of the daily CTV with respect to CTV_plan_. Because the posterior CTV margin is of particular interest regarding rectal toxicity, we separately evaluated the dislocation of the daily CTV contour towards the rectum by determining the absolute volume of each CTV_CBCTi_ outside of PTV^xmm^ in the overlap region between CTV_CBCTi_ and the rectum contour expanded by 5 mm. The corresponding Hausdorff variable was named HD^post^ (superscript post: posterior).

For every patient, several points of interest were defined: one point at the center of CTV_plan_ (P_0_), 4‐6 points at the posterior periphery of the prostate or in the seminal vesicle region (P_i_, i = 1,…,6), plus the point in CTV_plan_ causing the maximum HD^post^‐value over all fractions (denoted by P_worst_). In each case, a small 3D structure was contoured in Eclipse whose center‐of‐mass defined the point. The points of interest were propagated to each CBCT_i_ using the calculated deformable image registrations. The deformation vector components in left‐right direction (x‐component), anterior‐posterior direction (y‐component), and superior‐inferior direction (z‐component) at these points were analyzed.

PTV margins were calculated using the recipe proposed by van Herk^15^: To ensure a minimum dose to the CTV of 95% for 90% of the patients, a CTV‐to‐PTV margin of 2.5·Σ+1.64·σ’–1.64·σ_p_ is required, where Σ denotes the total standard deviation (SD) of preparation (systematic) errors, σ’ the total SD of execution (random) errors combined with the penumbra width, and σ_p_ the SD describing the penumbra width, and σ’^2^ = σ+σ_p_^2^.

#### 2.B.2 Dosimetric evaluations

Because the workflow for accumulation of full dose distributions over a treatment series is not supported by Eclipse, the dose delivered to selected points of interest per fraction was determined to enable dose summation at these points. For that purpose, the fraction dose at each point was derived from the dose at its displaced position obtained with the calculated deformation vector field. All dose values were normalized to the planned dose at the respective point (denoted by D_rel_). Point P_worst_ was analyzed for all 24 patients, points P_0_ and P_i_ were investigated for the four patients with the worst HD^post^ distributions (called worst HD^post^ patients). Posterior dose gradients were measured in each patient's treatment plan from the PTV periphery up to 7 mm towards the posterior rectal wall, at a height midway between base and apex of the prostate CTV.

To facilitate full dose accumulation over the treatment series, 15 patients were transferred to the software package MIM (V6.9.6, MIM Software Inc., Cleveland, OH, USA), which allows dose accumulation based on the planned dose distribution and the deformable image registrations calculated in Eclipse. For data selection, patients were ranked according to the maximum HD^post^ value in their treatment series and the 50%–95% quantiles of the HD^post^ distribution. All eight patients in the worst‐performing third were selected plus 5/8 and 2/8 randomly selected patients in the intermediate‐performing and best‐performing third, respectively, yielding 15 patients with 454 CBCTs. Within MIM, dose accumulation over the series of CBCTs was performed assuming the validity of the static dose cloud approximation.^16^ The planned dose distribution was first rigidly copied to each CBCT_i_ using the Eclipse online match and then deformably warped back to the planning CT using the deformable image registrations calculated in Eclipse. Subsequently, the dose contributions from each CBCT_i_ were summed up in the planning CT. Dose volume histograms (DVH) of CTV_plan_ for the original clinical dose distribution and the accumulated dose distribution accounting for deformations over the treatment series as well as CTV_CBCTi_‐DVHs of the individual fractions were exported for further analysis.

In addition to the clinical plans with 6 mm posterior margin, supplementary plans based on an isotropic 2 mm CTV‐to‐PTV margin were retrospectively optimized for the same treatment technique and constraints as the clinical plans and analyzed to investigate the effect of a reduced margin. This study included 12 patients with 364 CBCTs (8/2/2 patients in the worst‐/ intermediate‐/best‐performing third, respectively).

To quantify dosimetric consequences of any dose deviations from the planned dose distribution, the concept of equivalent uniform dose (EUD)^17^ was applied using two different approaches: (a) The original EUD model^17^ based on clonogen survival (EUD_SF_) with parameter values SF_2_ = 0.6 for the surviving fraction of clonogenic cells at 2 Gy and α/β = 2 Gy for the fractionation sensitivity of prostate cancer.^18^, ^19^ This was equivalent to a tumor control probability of 80% for a tumor consisting of 10^8^ clonogenic cells^20^ irradiated to a total dose of 78 Gy delivered with 2 Gy per fraction. For the EUD calculations, the dose distributions were normalized to 78 Gy (2 Gy per fraction) at the prescription point. (b) The phenomenological power law model as generalized concept of EUD (gEUD)^21^, ^22^ with two choices of the tissue‐specific parameter a = −7 (gEUD_a=−7_) and a = −20 (gEUD_a=−20_).^23^ EUD values were determined for planned (${\text{EUD}}_{\text{SF}}^{\text{plan}}$, ${\text{EUD}}_{a}^{\text{plan}}$), daily (${\text{EUD}}_{\text{SF}}^{\text{ind}}$, ${\text{EUD}}_{a}^{\text{ind}}$), and accumulated dose distributions (${\text{EUD}}_{\text{SF}}^{\text{accum}}$, ${\text{EUD}}_{a}^{\text{accum}}$), respectively.

#### 2.B.3 Time series analyses

To investigate the variation of daily residual deviations from the treatment plan as function of time over the course of treatment, time series analyses were performed. Both spatial (posterior displacement of point P_worst_, HD^post^) and dosimetric (D_rel_ at P_worst_) variables were evaluated for the four worst HD^post^ patients. The time series analyses were performed in SAS (version 14.1, SAS Institute, Cary, NC, USA). Nonstationary or random walk was tested using an augmented Dickey‐Fuller unit root test based on an autoregressive model with a single mean and time trend (rho statistic). To search for any similarity between observations as a function of the time lag between them, positive or negative first to fourth order autocorrelation in the time series was analyzed by means of the Durban‐Watson statistic (Proc AUTOREG). Bartlett's Kolmogorov‐Smirnov test statistic was applied to test the white noise hypothesis (Proc SPECTRA). Outliers (i.e. shock signatures) in the time series which could not be accounted for by the estimated model were detected using an autoregressive integrated moving average (ARIMA) model of fourth order at α = 0.001 (Proc ARIMA).

### 2.C Statistical analysis

Statistical analyses were performed in SAS (version 14.1, SAS Institute, Cary, NC, USA) and SPSS Statistics (version 22, IBM, Armonk, NY, USA). All statistical tests and procedures used are specified together with the results. P‐values were two‐sided, and *P* < 0.05 was considered statistically significant.

## 3 RESULTS

### 3.A Size of planned and accumulated volumes

For the 24 patients, the average size of CTV_plan_, CTV_acc_, and PTV amounted to 91.5 ± 6.2 cm^3^ (mean ± S.E.M.), 154.7 ± 10.5 cm^3^, and 237.9 ± 10.9 cm^3^, respectively. On average, CTV_acc_ was larger by a factor of 1.73 ± 0.06 than CTV_plan_. The PTV was larger by a factor of 1.61 ± 0.07 than CTV_acc_. An average CTV_CBCTi_ volume of 3.13 ± 0.19 cm^3^ (0.84 ± 0.09 cm^3^) was per fraction outside of PTV^2mm^ (PTV^5mm^). In the posterior direction, an average fractional CTV_CBCTi_ volume of 1.26 ± 0.10 cm^3^ (0.36 ± 0.05 cm^3^) was outside of PTV^2mm^ (PTV^5mm^).

RT_postop_ patients had significantly larger CTV_plan_ and CTV_acc_ volumes than RT_def_ patients. Also the CTV_CBCTi_ volume outside of PTV^2mm^ (PTV^5mm^) was larger, both isotropically and in posterior direction (cf. comparison of RT_def_ and RT_postop_ patients in Table S1). PTV size, bladder and endorectal balloon volumes were not different in the two patient groups.

### 3.B Spatial considerations

**FIG. 1:**
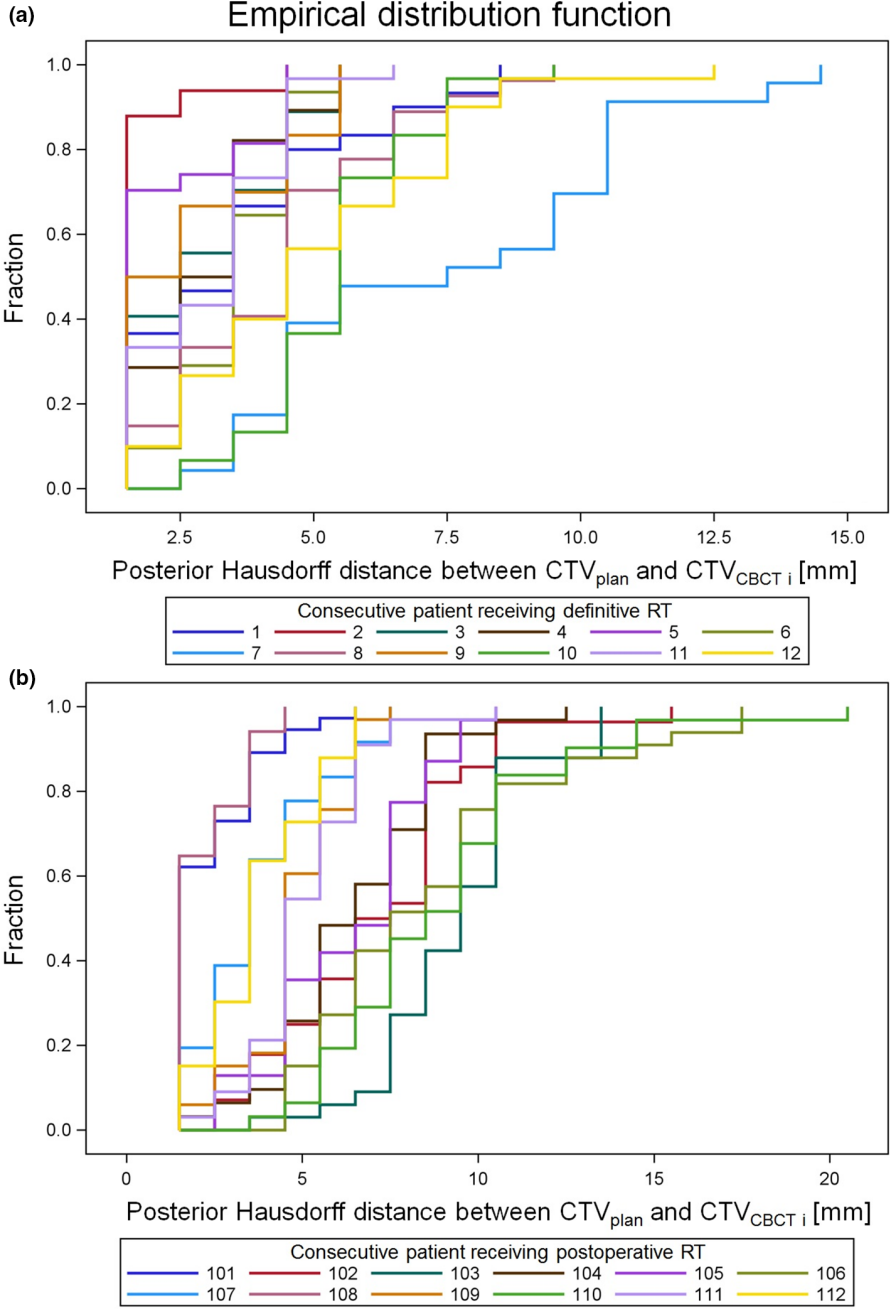
Cumulative distribution functions of the posterior Hausdorff distance HD^post^ between the CTV contour in the treatment plan (CTV_plan_) and the CTV contour in the i^th^ CBCT (CTV_CBCTi_) for patients treated with definitive (a) or postoperative (b) radiotherapy, respectively. At any specified value of the Hausdorff distance, the fraction of treatment sessions with a measured Hausdorff distance less than or equal to the specified value is plotted.

**FIG. 2:**
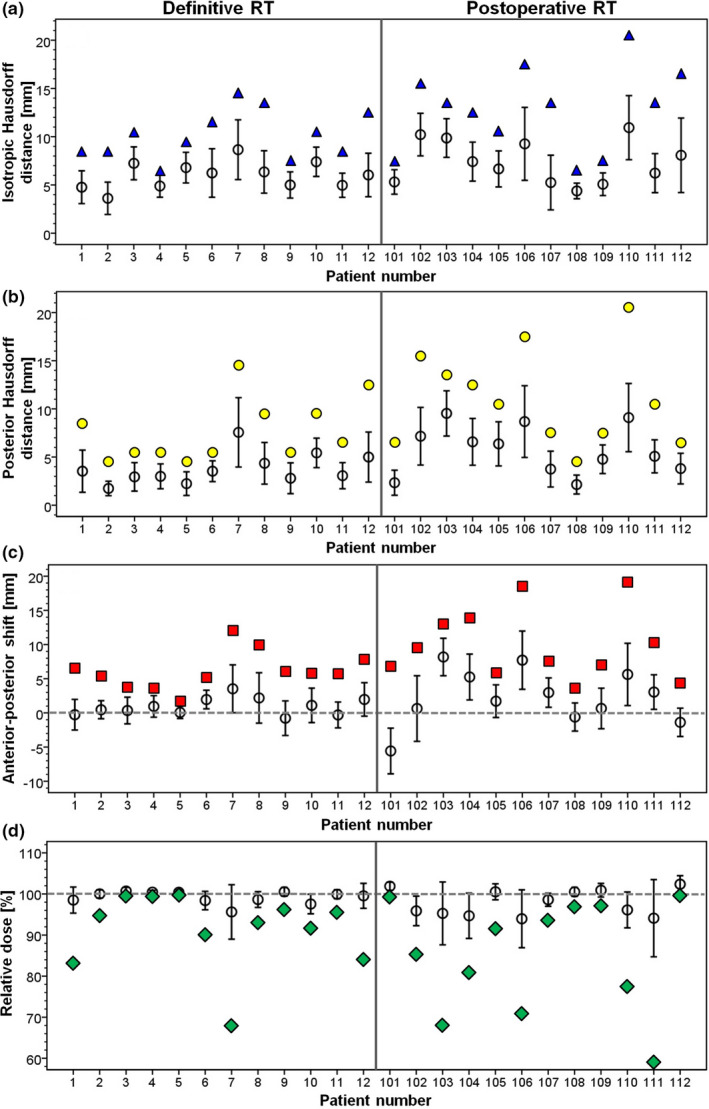
(a) Isotropic and (b) posterior Hausdorff distance, (c) anterior‐posterior shift assessed using the y‐component of the deformation vector, and (d) relative dose for prostate patients treated with definitive (patients 1‐12) and postoperative (patients 101‐112) radiotherapy, respectively. For every patient, the anterior‐posterior shift and the relative dose were determined at point P_worst_ which caused the largest posterior Hausdorff distance over all fractions. In each plot, the black circles with error bars indicate mean value ± one standard deviation of the distribution over the treatment series. The colored symbols show the extreme values in the distributions (i.e. the maximum for isotropic and posterior Hausdorff distance and anterior‐posterior shift; the minimum for relative dose).

Empirical distribution functions of HD^post^ determined for every fraction of the RT_def_ and RT_postop_ patients are shown in Figs. 1(a) and 1(b), respectively. Figures S1(a)–S1(d) illustrate the variation of HD^iso^ and HD^post^ over the treatment series for each individual RT_def_ and RT_postop_ patient. The mean and maximum values of the HD^iso^ and HD^post^ distributions per patient are shown in Figs. 2(a) and 2(b) and summarized in Table 1. Additional parameters characterizing the distributions including 50% and 95% quantiles are provided in Table S2. A significant interpatient variability in both the HD^iso^ and HD^post^ distributions among all patients was observed (*P* < 0.0005, Kruskal‐Wallis test). The median HD^iso^ values (HD^post^ values) for RT_def_ and RT_postop_ patients amounted to 5.5 mm and 6.5 mm (3.5 mm and 5.5 mm), respectively. The patients with largest 50%‐95% quantiles of the HD^post^ distribution over the treatment series were RT_def_‐patient 7 and RT_postop_ patients 103, 106, and 110 (denoted as worst HD^post^ patients).

**TABLE 1:** Parameters summarizing the distributions of the Hausdorff distance, the anterior‐posterior deformation vector component y and the relative dose at point P_worst_ over the treatment series as well as EUD_SF_ at P_worst_ for prostate patients treated with definitive (patients 1–12) or postoperative radiotherapy (patients 101–112), respectively.

		Hausdorff distance						Deformation vector at P_worst_			Relative dose at P_worst_			
		Distribution of HD^iso^ over all fractions			Distribution of HD^post^ over all fractions			Distribution of y over all fractions			Distribution over all fractions			
Pat	No of	HD_max_^iso^	Mean	Sigma	HD_max_^post^	Mean	Sigma	Mean	Sigma	Max	Mean	Sigma	Min	EUD_SF_
No	CBCTs	[mm]	[mm]	[mm]	[mm]	[mm]	[mm]	[mm]	[mm]	[mm]	[%]	[%]	[%]	[Gy]
1	30	8.5	4.8	1.7	8.5	3.5	2.2	−0.26	2.24	6.6	98.52	3.17	83.13	2.056
2	33	8.5	3.6	1.7	4.5	1.7	0.8	0.47	1.32	5.3	100.01	1.02	94.64	2.054
3	27	10.5	7.2	1.7	5.5	2.9	1.5	0.35	1.95	3.8	100.71	0.89	99.39	1.990
4	28	6.5	4.9	1.2	5.5	3.0	1.3	0.95	1.58	3.6	100.43	0.45	99.31	2.051
5	27	9.5	6.8	1.6	4.5	2.2	1.2	0.07	0.89	1.7	100.42	0.56	99.49	1.958
6	31	11.5	6.2	2.5	5.5	3.5	1.1	1.96	1.36	5.1	98.40	2.25	90.07	1.963
7	23	14.5	8.7	3.1	14.5	7.6	3.6	3.53	3.50	12.0	95.63	6.63	67.76	1.931
8	27	13.5	6.4	2.2	9.5	4.4	2.2	2.19	3.69	9.9	98.66	1.92	92.94	1.985
9	30	7.5	5.0	1.4	5.5	2.8	1.6	−0.77	2.53	6.0	100.53	1.05	96.09	2.007
10	30	10.5	7.4	1.5	9.5	5.4	1.5	1.10	2.52	5.8	97.55	2.37	91.57	1.944
11	30	8.5	5.0	1.3	6.5	3.1	1.4	−0.30	1.89	5.7	99.93	1.10	95.53	2.013
12	30	12.5	6.0	2.3	12.5	5.0	2.6	1.97	2.45	7.8	99.55	3.04	84.03	1.996
101	37	7.5	5.3	1.3	6.5	2.3	1.3	−5.57	3.34	6.8	101.89	1.00	99.29	1.997
102	28	15.5	10.2	2.2	15.5	7.2	3.0	0.64	4.80	9.5	95.88	3.59	85.31	1.907
103	33	13.5	9.9	2.0	13.5	9.5	2.3	8.18	2.74	13.0	95.28	7.65	68.14	1.852
104	31	12.5	7.4	2.0	12.5	6.6	2.4	5.25	3.35	13.9	94.69	5.51	80.83	1.909
105	31	10.5	6.7	1.9	10.5	6.4	2.3	1.72	2.39	5.9	100.53	1.95	91.48	2.007
106	33	17.5	9.3	3.8	17.5	8.7	3.7	7.72	4.26	18.5	93.96	7.05	70.68	1.862
107	36	13.5	5.3	2.8	7.5	3.8	1.9	2.97	2.15	7.6	98.59	1.59	93.71	1.944
108	34	6.5	4.4	0.8	4.5	2.1	1.0	−0.60	2.06	3.7	100.51	1.10	98.37	1.974
109	33	7.5	5.1	1.2	7.5	4.8	1.5	0.65	2.97	7.0	100.91	1.66	98.77	1.974
110	31	20.5	10.9	3.3	20.5	9.1	3.5	5.63	4.56	19.1	96.12	4.37	77.27	1.862
111	33	13.5	6.2	2.0	10.5	5.1	1.7	3.05	2.53	10.3	94.11	9.40	59.09	1.952
112	33	16.5	8.1	3.9	6.5	3.8	1.6	−1.38	2.07	4.3	102.38	2.08	99.38	1.986

Abbreviations: EUD_SF,_ equivalent uniform dose calculated using a cell survival model; HD_max_, maximum value of the Hausdorff distance between planned and daily CTV over all treatment fractions per patient; iso, isotropic; Positive y‐value, posterior shift; post, posterior.

Table S3 lists the number of fractions in which selected spatial parameters exceeded a given threshold value and quotes across how many patients these fractions were distributed. For example, large HD^iso^ values ≥12.5 mm (≥15 mm) were observed in 37/739 (9/739) fractions distributed across 11 (4) patients. Large HD^post^ values ≥12.5 mm (≥15 mm) occurred in 20/739 (5/739) fractions distributed across 7 (3) patients.

For all RT_def_ patients, point P_worst_ which caused the maximum HD^post^ value over all fractions was located at the posterior CTV boundary, either in the seminal vesicle region (5/12 patients, 2/5 patients with HD^post^ ≥ 12.5 mm), close to the apex (3/12 patients, no patient with HD^post^ ≥ 12.5 mm) or in an intermediate position (4/12 patients, no patient with HD^post^ ≥ 12.5 mm). For 11/12 RT_postop_ patients (4/11 patients with HD^post^ ≥ 12.5 mm), P_worst_ was located in the seminal vesicle region. For 1/12 RT_postop_ patient (HD^post^ ≥ 12.5 mm), P_worst_ was between prostate apex and base.

The anterior‐posterior shift at point P_worst_ for every fraction of RT_def_ and RT_postop_ patients is displayed in Figs. S2(a) and S2(b), respectively. The mean and maximum values of each patient's distribution of the anterior‐posterior shift at point P_worst_, assessed using the y‐component of the deformation vector at that point, are visualized in Fig. 2(c) and listed in Table 1. Table S4 provides corresponding numbers for the shifts in left‐right direction (x‐component of the deformation vector) and superior‐inferior direction (z‐component) as well as additional parameters characterizing the distributions. Shifts of point P_worst_ in x, y, and z were weakly correlated over all patients (correlation of y with x: Spearman correlation coefficient r_s_ = 0.14, *P* = 0.0001; correlation of y with z: r_s_ = −0.15, *P* = 0.0001). Deviations in y were larger than in x or z (mean values over all patients and fractions: x = 0.78 mm, SD = 2.59 mm; y = 1.62 mm, SD = 4.10 mm; z = −0.45 mm, SD = 3.35 mm).

**FIG. 3:**
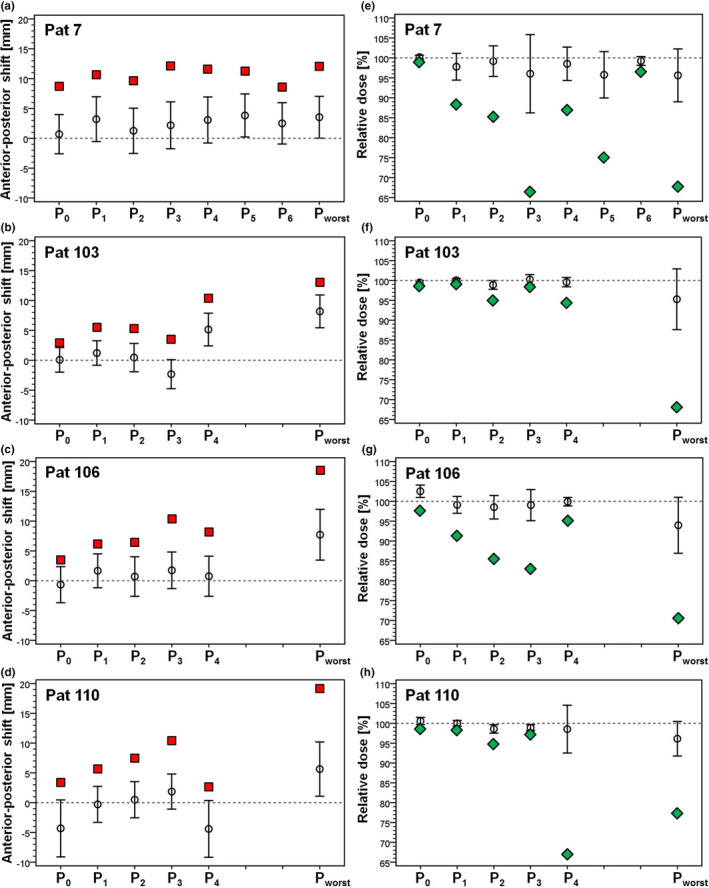
(a–d) Anterior‐posterior shift and (e–h) relative dose at different points of interest within the planned CTV for the four prostate patients with worst distribution of the posterior Hausdorff distance over the treatment series. In each plot, the black circles with error bars represent mean value ± one standard deviation of the distribution. The red squares indicate the maximum posterior shift at each point. The green symbols visualize the minimum values of the relative dose distributions. The dashed horizontal lines correspond to the undisturbed values of the treatment plan.

Figures 3(a)–3(d) display the main characteristics of the distributions of the anterior‐posterior shift at all specified points of interest in CTV_plan_ over the treatment series for the four worst HD^post^ patients. The numbers are summarized in Table S5. Generally not only the maximum values but also the mean posterior shifts were larger at point P_worst_ than at the other points of interest in the respective patient, except for patient 7, points P_3_ and P_5_.

Margin calculations^15^ using Σ = 2.98 mm (SD of mean values per patient), σ = 2.76 mm (SD of y per patient around the mean), and σ_p_ = 4.12 mm (cf. section 3.C. Dosimetric considerations), hence σ’ = 4.96 mm, derived from our data, yielded a posterior PTV margin of 8.8 mm (9.7 mm) required to ensure a minimum dose in the CTV of 95% for 90% (95%) of our patients.

### 3.C Dosimetric considerations

Figures S2(c) and S2(d) visualize the relative dose at point P_worst_ for every treatment fraction of the RT_def_ and RT_postop_ patients, respectively. Characteristics of each patient's D_rel_ distribution over the treatment series are illustrated in Fig. 2(d) and included in Table 1. Also quoted are EUD_SF_ values which, when constantly applied over the treatment course, would lead to the same cell kill as the actually delivered, from fraction to fraction varying, dose. EUD_SF_ at P_worst_ did not drop below 1.85 Gy per fraction for any of the patients. For 21/24 patients (87.5%), the decline in EUD_SF_ with respect to the planned value was <5%.

Figures 3(e)–3(h) present the main parameters of the D_rel_ distributions at all specified points of interest within CTV_plan_ over the treatment series for the four worst HD^post^ patients. Corresponding parameters describing the distributions of dose deviations from the treatment plan over the series are listed in Table S5. For every patient, a significant heterogeneity in the D_rel_ distribution from point to point was observed, indicating that the expectation value for the accumulated dose is not equal at all points.

**FIG. 4:**
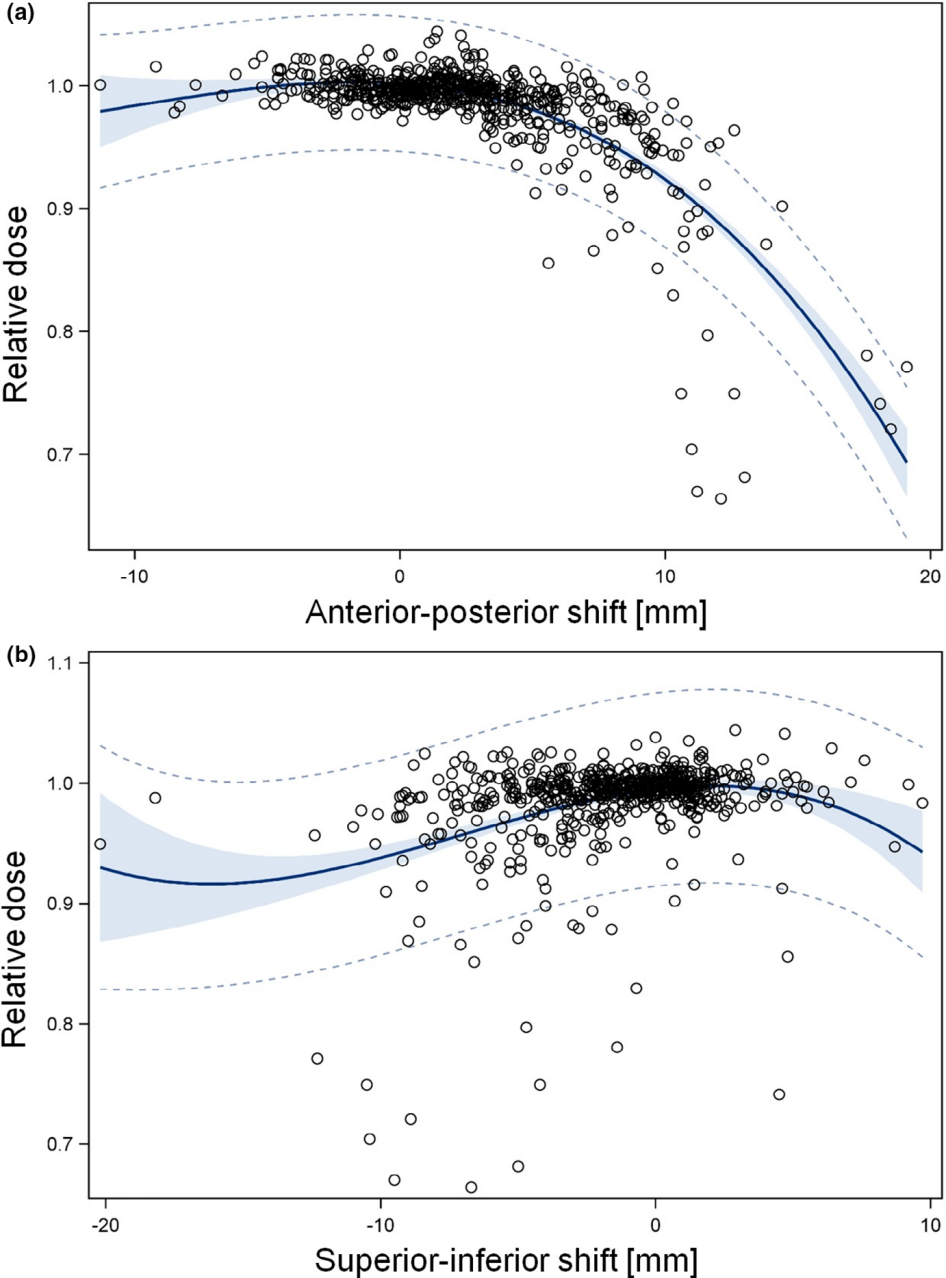
Scatter plots of the relative dose at selected points of interest in the planned CTV plotted versus (a) the anterior‐posterior and (b) the superior‐inferior shift, assessed by the y‐ and z‐components, respectively, of the deformation vector at the considered point for all 120 fractions of the four worst HD^post^ patients (patients 7, 103, 106, and 110). Only the points of interest at the posterior periphery of the CTV or in the seminal vesicle region (P_i_) and point P_worst_ were included. The points P_0_ located at the center of the prostate were excluded, yielding 646 entries per plot. Results of the fit of a third degree polynomial to the data are also shown (Solid blue line: fit result; dashed line: 95% prediction limit; area shaded in blue: 95% confidence limits). All terms up to n = 3 were significant.

To investigate dosimetric consequences of daily residual distortions, the dependence of D_rel_ on anterior‐posterior and superior‐inferior shifts, assessed through the y‐ and z‐components, respectively, of the deformation vector at selected points of interest was evaluated. Regression analysis of all 120 fractions of the four worst HD^post^ patients revealed a substantially stronger dependence of D_rel_ on anterior‐posterior shifts compared with superior‐inferior shifts (Fig. 4). With increasing posterior shifts, a pronounced drop in D_rel_ was observed, consistent with the expected decline due to the dose gradient measured in each patient's treatment plan. The posterior dose gradient averaged over all 24 treatment plans amounted to 4.21 ± 0.10% per mm and could be approximated by a gaussian distribution with σ_p_ = 4.12 mm. Dose gradients for RT_def_ and RT_postop_ patients were not different (*P* = 0.07, F‐test).

Table 2 lists absolute EUD_SF_ and gEUD values calculated using a = −20 and a = −7 for the original and accumulated dose distributions of the clinical 6 mm plans for 15 patients. Resulting percent EUD differences between original and accumulated dose distributions are included. For the clinical 6‐mm plans, the deteriorations in EUD_SF_ were smaller than 5% in 14/15 patients (93.3%). Only 1/15 patient experienced a decline in EUD_SF_ of 5.4%. The drop in gEUD_a=−20_ was smaller than 5% for all 15 patients. As expected, the decline in gEUD_a=−7_ was always smaller than that in gEUD_a=−20_, since the parameter value a=−7 makes gEUD less sensitive to cold spots in the dose distributions. In general, a strong correlation between EUD_SF_ and gEUD_a=−20_ was observed (12 patients with 364 fractions: Pearson correlation coefficient r_P_ = 0.986, *P* < 0.0005; Spearman correlation coefficient r_S_ = 0.985, *P* < 0.0005). The correlation between EUD_SF_ and gEUD_a=−7_ was weaker, yet statistically significant (r_P_ = 0.722, *P* < 0.0005; r_S_ = 0.717, *P* < 0.0005).

**TABLE 2:** Absolute EUD_SF_‐ and gEUD‐values for a = −20 and a = −7 of the original and accumulated dose distributions and resulting EUD differences for clinical treatment plans (6 mm PTV‐margin) and plans with 2 mm PTV‐margin.

Pat no	Original dose distribution			Accumulated dose distribution			Difference (original‐accumulated)		
	EUD_SF_ [Gy]	gEUD_a=−20_ [Gy]	gEUD_a=−7_ [Gy]	EUD_SF_ [Gy]	gEUD_a=−20_ [Gy]	gEUD_a=−7_ [Gy]	Δ EUD_SF_ [%]	Δ gEUD_a=−20_ [%]	Δ gEUD_a=−7_ [%]
6 mm‐margin (clinical plans)									
1	78.66	78.75	78.88	78.83	78.88	78.96	−0.22	−0.17	−0.10
5	78.25	78.32	78.42	78.52	78.55	78.60	−0.35	−0.30	−0.23
7	78.41	78.44	78.49	77.90	77.97	78.06	0.66	0.61	0.54
8	78.42	78.47	78.55	78.47	78.49	78.53	−0.06	−0.03	0.02
10	78.95	79.01	79.08	79.02	79.06	79.10	−0.09	−0.06	−0.03
12	79.78	79.83	79.90	79.88	79.90	79.93	−0.13	−0.09	−0.03
102	78.69	78.81	78.99	78.18	78.35	78.59	0.66	0.58	0.51
103	79.04	79.19	79.41	77.25	78.02	79.00	2.26	1.47	0.52
104	79.60	79.77	80.00	75.27	76.57	79.22	5.43	4.01	0.97
105	78.80	78.88	78.98	78.89	78.92	78.98	−0.10	−0.06	0.00
106	79.06	79.16	79.31	78.20	78.42	78.72	1.10	0.93	0.74
108	78.40	78.49	78.62	78.62	78.66	78.72	−0.27	−0.22	−0.13
109	78.76	78.87	79.04	78.89	78.97	79.08	−0.16	−0.12	−0.05
110	78.30	78.41	78.59	78.06	78.20	78.41	0.31	0.27	0.23
111	78.85	78.95	79.09	78.46	78.65	78.89	0.50	0.38	0.25
2 mm‐margin									
1	78.66	78.74	78.85	76.90	77.54	78.36	2.25	1.52	0.63
5	78.72	78.77	78.85	77.60	77.97	78.38	1.42	1.02	0.60
7	78.48	78.52	78.58	74.23	74.82	76.07	5.41	4.72	3.19
12	78.73	78.76	78.81	77.50	77.74	78.04	1.56	1.29	0.98
102	79.07	79.15	79.26	75.36	76.21	77.73	4.69	3.71	1.93
103	79.25	79.45	79.74	66.19	63.59	77.13	16.48	19.96	3.28
104	78.65	78.77	78.96	75.34	76.33	78.10	4.20	3.10	1.10
105	78.53	78.59	78.67	69.04	69.06	76.40	12.09	12.12	2.89
106	78.77	78.84	78.94	73.47	74.16	77.18	6.73	5.93	2.23
108	78.79	78.86	78.96	78.47	78.59	78.73	0.41	0.34	0.28
110	78.35	78.42	78.53	74.62	75.23	76.45	4.77	4.07	2.65
111	78.21	78.33	78.51	70.21	70.82	75.88	10.22	9.59	3.35

Abbreviations: EUD, equivalent uniform dose; EUD_SF_, EUD calculated using a cell survival model; gEUD, generalized concept of EUD.

Corresponding values of EUD_SF_, gEUD_a=−20_, and gEUD_a=−7_ for the original and accumulated dose distributions of 12 plans with 2‐mm margin are included in Table 2. In total, 3/12 patients (25%) experienced a decline in EUD_SF_ of >10% between original and accumulated dose distributions. In 7/12 patients (58.3%), EUD_SF_ deteriorated by <5%.

**FIG. 5:**
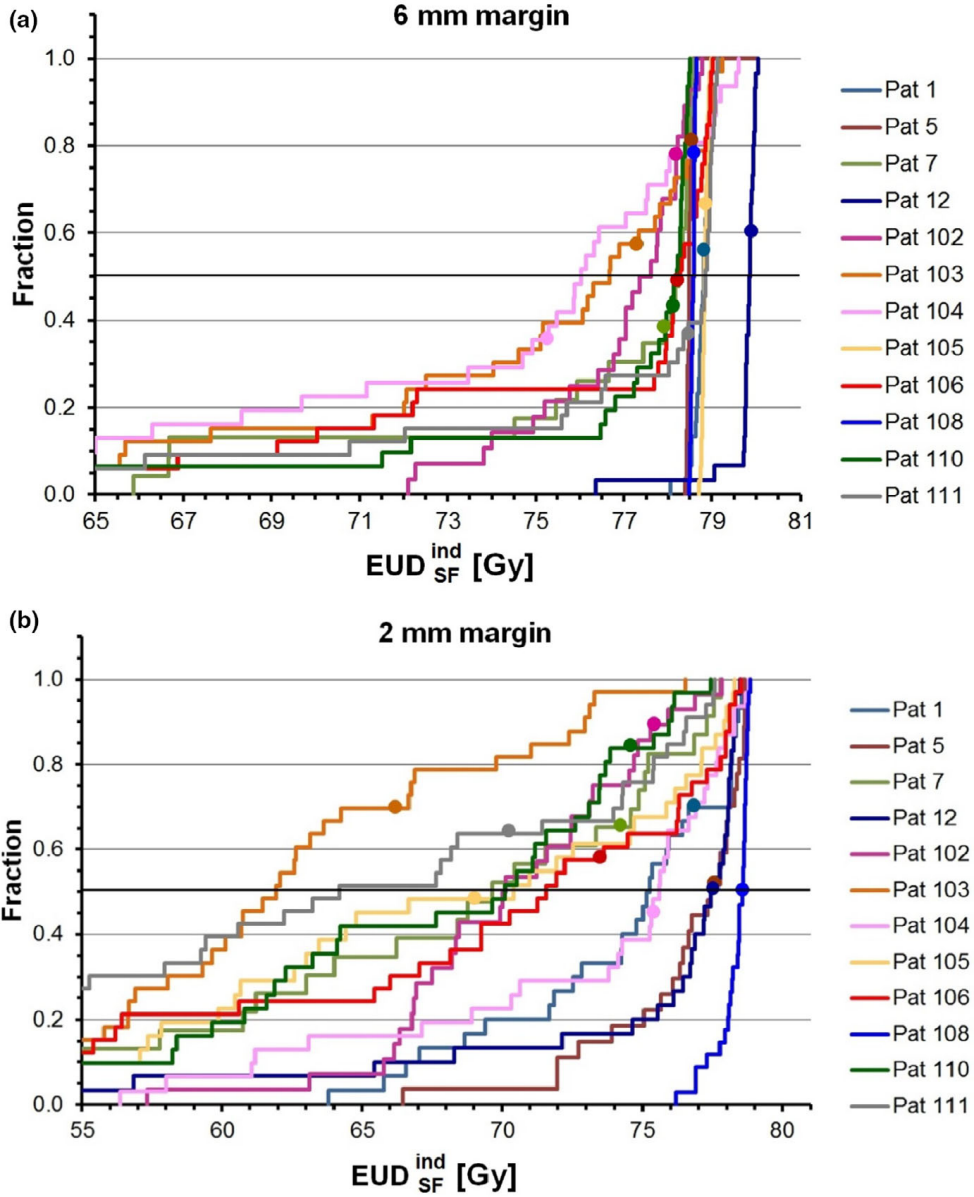
Cumulative distribution functions of the equivalent uniform dose calculated for each individual fraction (${\text{EUD}}_{\text{SF}}^{\text{ind}}$) of 12 prostate patients. At any specified value of ${\text{EUD}}_{\text{SF}}^{\text{ind}}$, the fraction of treatment sessions with a measured ${\text{EUD}}_{\text{SF}}^{\text{ind}}$ less than or equal to the specified value is plotted. (a) Clinical plans with 6‐mm margin. (b) Plans with 2‐mm margin. For every patient, the EUD value of the dose distribution accumulated over all treatment fractions (${\text{EUD}}_{\text{SF}}^{\text{accum}}$) is indicated by a circle.

**FIG. 6:**
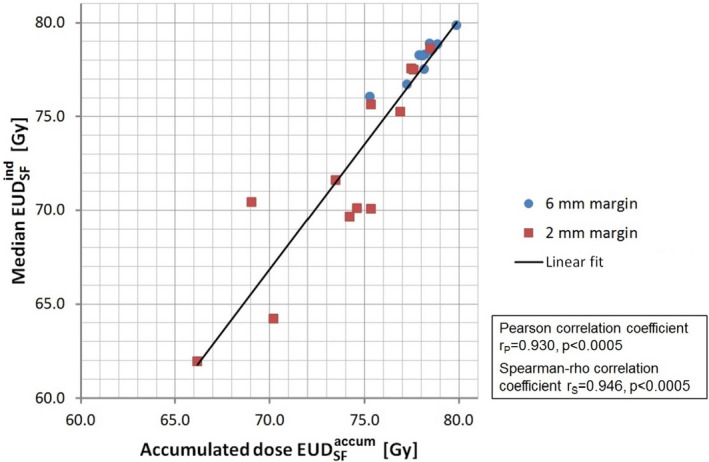
Scatter plot of the median value of the distribution of the equivalent uniform dose values calculated for every treatment fraction of each patient (${\text{EUD}}_{\text{SF}}^{\text{ind}}$) versus the equivalent uniform dose derived from the accumulated dose distribution (${\text{EUD}}_{\text{SF}}^{\text{accum}}$). The plot includes 6‐mm and 2‐mm plans for 12 patients.

Figure 5(a) shows cumulative distribution functions of ${\text{EUD}}_{\text{SF}}^{\text{ind}}$ calculated for each individual treatment fraction of the clinical 6‐mm plans for 12 patients (4 RT_def_, 8 RT_postop_). For every patient, the ${\text{EUD}}_{\text{SF}}^{\text{accum}}$ value derived from the accumulated dose distribution is indicated by a circle. For better resolution in the high‐dose region, the low‐dose part was omitted. The cumulative distribution function with full scale is shown in Fig. S3(a). Figure 5(b) and Figure S3(b) show the corresponding plot for the 2‐mm plans of the same patients. The ${\text{EUD}}_{\text{SF}}^{\text{accum}}$ values cluster around the median values of the ${\text{EUD}}_{\text{SF}}^{\text{ind}}$‐distributions. In Fig. 6, the median of each patient's ${\text{EUD}}_{\text{SF}}^{\text{ind}}$‐distribution is plotted versus the ${\text{EUD}}_{\text{SF}}^{\text{accum}}$ value for both 6‐mm and 2‐mm plans. Corresponding plots based on gEUD_a=−20_ and gEUD_a=−7_ are shown in Figs. S4(a) and (b), respectively. A strong correlation between the median ${\text{EUD}}_{\text{SF}}^{\text{ind}}$ value and ${\text{EUD}}_{\text{SF}}^{\text{accum}}$ was observed (r_P_ = 0.930, *P* < 0.0005; r_S_ = 0.946, *P* < 0.0005), indicating that ${\text{EUD}}_{\text{SF}}^{\text{accum}}$ derived from the accumulated dose distribution could well be approximated by the median ${\text{EUD}}_{\text{SF}}^{\text{ind}}$ value for every plan.

The total dose at point P_worst_ derived from the full accumulated dose distributions of all 6‐mm and 2–mm plans was between the minimum dose D_min_ and D_99_ for 15/27 = 55.6%, between D_min_ and D_98_ for 22/27 = 81.5%, and between D_min_ and D_97_ for 25/27 = 92.6% of the plans, respectively, indicating that P_worst_ received a dose close to the minimum value in the accumulated dose distribution.

### 3.D Time series

**FIG. 7:**
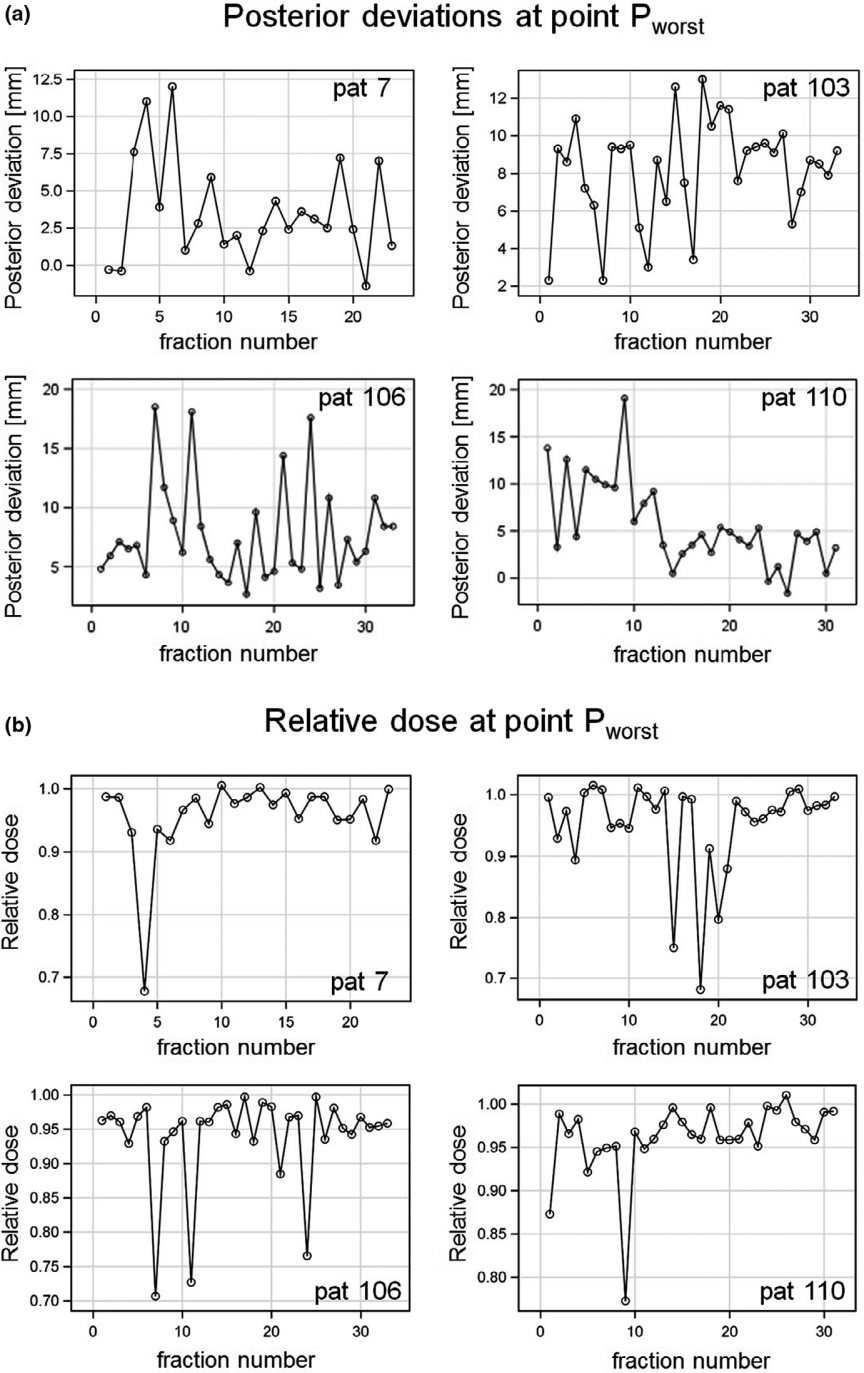
Time series plots for the four patients with largest HD^post^ dispersion over all treatment fractions. (a) Posterior deviations and (b) relative dose, respectively, at point P_worst_.

The time series of posterior shifts at point P_worst_ over all fractions are shown in Fig. 7(a) for the four worst HD^post^‐patients. For all patients, nonstationary or random walk could be rejected (Dickey‐Fuller unit root test, rho statistic, *P* < 0.005). For patients 7, 103, and 106, there was no positive or negative first to fourth order autocorrelation of the posterior deviations around their mean with preceding values in the series (Durban‐Watson statistic, Proc AUTOREG, SAS). For patient 110, a positive second order autocorrelation was found (Durbin‐Watson statistic, *P* = 0.0002). The white noise hypothesis could not be rejected in any of the patients (Bartlett's Kolmogorov‐Smirnov test, *P* > 0.50, Proc SPECTRA, SAS). An additive shock signature in the time series was identified at fraction 9 of patient 110 (Proc ARIMA, SAS).

Very similar results were derived for the time series of HD^post^ (Fig. S5), with the exception that no autocorrelation was found for patient 110. Specifically, nonstationary or random walk could be rejected for all four patients (Dickey‐Fuller unit root test, rho statistic, *P* < 0.015), whereas the white noise hypothesis could not be rejected in any of the patients (Bartlett's Kolmogorov‐Smirnov test, *P* > 0.15). An additive outlier was also observed in the posterior Hausdorff distances at fraction 9 of patient 110.

Figure 7(b) shows the time series of D_rel_ at point P_worst_ for the worst HD^post^ patients. Nonstationary or random walk could be rejected for all patients (Dickey‐Fuller unit root test, rho statistic, *P* < 0.015). No positive or negative first to fourth order autocorrelation of the D_rel_ deviations with preceding values in the series was observed (Durban‐Watson statistic). The white noise hypothesis could not be rejected in any of the patients (Bartlett's Kolmogorov‐Smirnov test, *P* > 0.25). Additive outliers (at *P* = 0.001) in D_rel_ at P_worst_ were observed for patient 7 at fraction 4, for patient 106 at fractions 7, 11, and 14, and for patient 110 at fractions 1 and 9, respectively. In addition, temporary level shifts of D_rel_ per fraction were detected for patient 103 beginning at fraction 18 and lasting for three fractions and for patient 110 beginning at fraction 24.

## 4 DISCUSSION

Daily residual deformations after image‐guided definitive and postoperative radiotherapy of prostate cancer with endorectal balloon were quantified using various spatial and dosimetric parameters, and analyzed regarding their effect on the actually delivered dose distribution.

One of the spatial variables used was the Hausdorff distance quantifying the maximum displacement between daily and planned CTV contour. The HD distributions over all fractions, both when analyzed isotropically and restricted to the posterior region, demonstrated a significant interpatient variability, with RT_postop_ patients performing worse than RT_def_ patients. Substantially large HD^iso^ and HD^post^ values in one or more fractions were observed for several patients (Table S3; Fig. S1). For the majority of the patients (5/12 RT_def_, 11/12 RT_postop_), the point P_worst_ inside CTV_plan_, which exhibited the largest displacement over all fractions, was located at the posterior CTV_plan_ boundary in the region of the seminal vesicles or seminal vesicle bed. This finding is consistent with other studies reporting larger deformations of the seminal vesicles compared to deformations of the prostate with respect to intraprostatic fiducial markers^24^ and the need for larger margins for seminal vesicle CTVs compared to prostate CTVs.^25^, ^26^, ^27^ Similar to the HD distributions, the posterior shift at P_worst_ showed considerably large values for individual fractions in some patients, whereas the majority of the patients was not affected [Table S3; Figs. S2(a) and S2(b)].

To assess dosimetric consequences of daily residual CTV distortions, the dependence of the relative dose at point P_worst_ and other points of interest located at the posterior CTV_plan_ boundary on the posterior shift at the respective point was analyzed for every fraction [Figs. S2(c) and S2(d); Fig. 4(a)]. A pronounced decrease in relative dose with increasing posterior shifts was observed, consistent with the expected decline due to the measured posterior dose gradients. Restricting the analysis to the daily dose contribution at discrete points of interest within CTV_plan_ provides a simple means to assess the dose sum at selected points without the need to perform a full dose accumulation. From the dose sum at P_worst_, an EUD_SF_ value was derived which, when constantly delivered over the series, led to the same cell kill as the sum of the actually delivered dose contributions. Even at P_worst_, EUD_SF_ did not drop below 1.85 Gy for any of the 24 patients and remained >95% of the planned value for 21/24 patients (Table 1).

For a subset of the patients, accumulation of full dose distributions was performed. To quantify the difference between planned and accumulated dose distributions, the EUD_SF_ model based on clonogen survival^17^ and the generalized gEUD model^21^, ^28^ with parameter values a = −20 and a = −7^23^ was used (Table 2). The extreme choice a = −20 overemphasizes any cold spots. However, EUD_SF_ values assuming SF_2_ = 0.6 and α/β = 2 Gy were generally very similar to gEUD‐values for a = −20. Analyzing the clinical plans with 6‐mm margin, the decline in EUD_SF_ between original and accumulated dose distributions was <5% in 14/15 patients (93.3%). Only one patient (patient 104) showed an EUD_SF_ drop of 5.4%. Hence, a posterior 6‐mm margin is sufficient to maintain EUD_SF_ above 95% in > 93% of our patients. This estimate is conservative, because particularly patients with poor HD^post^ distribution were selected for the dose accumulation analysis (patient 104 belongs to the worst third). Even for 2‐mm plans, EUD_SF_ deteriorated by <10% and <5% in 75% and 58.3% of the patients, respectively. Hence, the accumulated dose distributions turned out to be forgiving of cold spots in individual treatment sessions. Due to the daily deformation of the prostate and resulting shifts in position with respect to the planned geometry, the dose per fraction at every point in the prostate varies over time. Cold spots at the posterior CTV periphery move from fraction to fraction and are therefore washed out over the series. Note that point P_worst_ is determined by one single fraction experiencing the largest shift during the treatment series and certainly did not receive the minimum dose value at every fraction. Nonetheless, the accumulated dose at P_worst_ was between D_min_ and D_98_ (D_97_) for 81.5% (92.6%) of the analyzed patients, indicating that points P_worst_, which were identified by geometrical considerations, received a dose very close to the minimum value of the full accumulated dose distributions. These findings demonstrate that spatial and dosimetric results are consistent.

EUD_SF_ of the accumulated dose distribution can well be approximated by the median value of the distribution of the ${\text{EUD}}_{\text{SF}}^{\text{ind}}$ values determined per fraction (Figs. 5 and 6). An EUD analysis of individual fractions can therefore serve as simple alternative to assess the actually delivered dose if software for dose accumulation is not available.

Our analysis of interfraction time series data, based on both spatial and dosimetric variables, could exclude nonstationary or random walk and, instead, revealed a white noise characteristic. This finding is in contrast to the intrafraction prostate motion which has been reported to be a random walk.^29^, ^30^, ^31^ In general, no autocorrelation, i.e. no similarity between observations as a function of the time lag between them, was observed. Since the time series are stationary, the expectation value is constant over time. However, the white noise amplitude differs significantly from patient to patient. On average, two outliers not explained by normal theory were observed per patient. To our knowledge, this is the first study that analyzed interfractional time series in prostate cancer.

A number of previous studies analyzed dosimetric consequences of interfractional CTV displacements and deformations by means of dose accumulation to evaluate appropriate PTV margins, based on weekly MV‐CBCTs,^32^ daily kV‐CBCTs,^33^, ^34^, ^35^ helical MVCTs^36^ or repeat planning CTs.^25^, ^26^, ^37^, ^38^ Only in two studies, an endorectal balloon was used.^36^, ^37^ The majority of the studies analyzed patients treated with definitive RT. One study evaluated patients receiving adjuvant RT of the prostate bed after prostatectomy.^32^ While a 3‐4 mm posterior margin was considered as too small in some studies,^25^, ^32^, ^33^, ^35^ it was found adequate in others.^26^, ^36^, ^37^, ^38^ However, the direct quantitative comparison of the studies is hindered by the diverse dosimetric criteria which were used to judge target coverage as adequate and the different navigation techniques applied. Qin et al. used gEUD_a=−20_ as dosimetric criterion, as we did, and found that for 4/22 patients gEUD_a=−20_ for the accumulated dose was <95% of the planned gEUD_a=−20_ and fell below 90% for one patient. They concluded that a 3‐mm margin was not appropriate to compensate residual variations with a daily correction technique.^33^ However, none of the above studies investigated residual deviations after full 6‐dof IGRT with online correction of both translations and rotations. In contrast to other studies, we evaluated not only the accumulated dose distribution, but also individual treatment fractions to assess their dose contribution, because this may provide guidelines regarding online navigation and adaptation strategies.

For any dose accumulation study, the accuracy of the algorithm to calculate the deformable image registrations is a matter of concern. The Eclipse algorithm which we used was evaluated according to the recommendations of the AAPM Task Group No. 132.^39^ Moreover, the registration results have been visually inspected by an experienced radiation oncologist. Our particular setting is favorable because the low density inside the air‐filled endorectal balloon provides a strong contrast with respect to the surrounding soft tissue in planning CT and daily CBCTs which dominates the deformable registration in the posterior direction. The displacement of the location of the anterior endorectal balloon wall (which is closely followed by the posterior CTV boundary) in the planning CT and in the deformably registered CBCTs, as measured in the sagittal plane, was less than 1 mm. To investigate the quality of the deformable image registration results in soft tissue distant from the endorectal balloon, we used small calcifications in the prostate gland or surgical clips in case of postoperatively treated patient as internal landmarks. On purpose, the fractions with largest posterior Hausdorff distance were selected for this analysis. An average residual error of 1.7 mm between the landmarks identified on the planning CT and on the deformably registered CBCT was derived. This is an acceptable accuracy for the described usage.

According to the commonly used margin recipe proposed by van Herk,^15^ a posterior PTV margin of 8.8 mm (9.7 mm) is required to ensure a minimum dose to the CTV of 95% for 90% (95%) of our patients. This margin is markedly larger than the 6‐mm margin proven to be sufficient for our dataset. A combination of several factors may explain the discrepancy: Most importantly, our data showed that a criterion on the minimum dose is not mandatory to maintain the target EUD_SF_ within tight limits when reasonable cell survival model parameters are chosen. Moreover, time series analysis of the dose at point P_worst_ showed outliers not predicted by normal distributions. Hence, the prerequisites for the computation of the cumulative dose distribution underlying the margin recipe are not fulfilled.^15^ Van Herk's formula infers the PTV margin for the individual patient from random deviations of the target position and the scatter of the systematic mean deviations over all patients. However, we demonstrated that residual deviations quantified by various spatial and dosimetric parameters differ significantly from patient to patient (Table S3). Applying uniform margins, derived under the inclusion of worst case patients, to the population will be overly conservative for the majority of patients. Our analysis has shown that a 2‐mm margin is still sufficient to maintain EUD_SF_ > 90% (95%) in 75% (58.3%) of the patients. When daily image guidance is used, the systematic setup error derived from a patient population is not relevant for the individual patient, instead the daily individual systematic deviation matters. It is reasonable to determine the PTV margin for the 80%–90% of patients with smaller deformations and use larger margins with or without adaptive replanning for the remainder, as identified during the treatment series.

## 5 CONCLUSIONS

This analysis of definitive and postoperative radiotherapy of prostate cancer with endorectal balloon demonstrated that dose distributions accumulated over the treatment series are forgiving of considerable residual distortions observed at a frequency below 50% of the fractions. PTV margin calculations at point P_worst_ with largest posterior Hausdorff distance over all fractions are overly conservative because daily online 6‐dof IGRT corrects translational and rotational errors such that only residual deformations have to be considered. Studies of this nature help establish a rational basis for online navigation, margin optimization, and treatment adaptation strategies.

## CONFLICT OF INTEREST

No conflicts of interest.

## AUTHOR CONTRIBUTIONS

Sabine Levegrün and Martin Stuschke conceived and designed the study, collected and analyzed the data, and interpreted the results. Sabine Levegrün drafted the manuscript. Christoph Pöttgen, Konstantinos Xydis, Maja Guberina, and Jehad Abu Jawad contributed to the data acquisition and preparation. All authors revised the manuscript and approved the final version.

## Supporting information


**Fig. S1** Isotropic Hausdorff distance HD^iso^ determined for every treatment fraction of patients treated with definitive (a) or postoperative (b) radiotherapy, respectively. Posterior Hausdorff distance HD^post^ per fraction for patients treated with definitive (c) or postoperative (d) radiotherapy, respectively.
**Fig. S2** Shift in anterior‐posterior direction assessed through the y‐component of the deformation vector at point P_worst_ for every treatment fraction of patients treated with definitive (a) or postoperative (b) radiotherapy, respectively. Positive values indicate a posterior shift. Relative dose D_rel_ at point P_worst_ per treatment fraction for patients treated with definitive (c) or postoperative (d) radiotherapy, respectively.
**Fig. S3** Cumulative distribution functions of ${\text{EUD}}_{\text{SF}}^{\text{ind}}$ for 12 prostate patients. At any specified value of ${\text{EUD}}_{\text{SF}}^{\text{ind}}$, the fraction of treatment sessions with a measured ${\text{EUD}}_{\text{SF}}^{\text{ind}}$ less than or equal to the specified value is plotted. a) Clinical plans with 6 mm margin. b) Plans with 2 mm margin.
**Fig. S4** Scatter plots of the median value of the distribution of the ${\text{gEUD}}_{a}^{\text{ind}}$‐values calculated for every individual treatment fraction of each patient versus ${\text{gEUD}}_{a}^{\text{accum}}$ derived from the accumulated dose distributions. (a) Parameter a = −20. (b) Parameter a = −7. The plots include both 6 mm and 2 mm CTV‐to‐PTV margin plans for 12 patients.
**Fig. S5** Time series plots of the posterior Hausdorff distance over all treatment fractions for the four patients with worst HD^post^ ‐distribution (patient 7: definitive radiotherapy, patients 103, 106, and 110: postoperative radiotherapy).

**Table S1** Comparison of prostate cancer patients treated with definitive and postoperative radiotherapy.
**Table S2** Parameters characterizing the distributions of the isotropic and posterior Hausdorff‐distances over the treatment series for prostate patients treated with definitive (patients 1‐12) or postoperative radiotherapy (patients 101‐112), respectively.
**Table S3** Number of fractions with selected spatial and dosimetric parameters exceeding a given threshold value. The numbers in brackets identify the corresponding patient(s).
**Table S4** Parameters characterizing the distributions of the deformation vector components over the treatment series for prostate patients treated with definitive (patients 1‐12) or postoperative radiotherapy (patients 101‐112).
**Table S5** Parameters summarizing the distributions of the anterior‐posterior deformation vector component y and of the dose deviations with respect to the treatment plan at different points of interest within the planned CTV.

## References

[1] Smeenk RJ , van Lin ENJ , van Kollenburg P , McColl GM , Kunze‐Busch M , Kaanders JHAM . Endorectal balloon reduces anorectal doses in post‐prostatectomy intensity‐modulated radiotherapy. Radiother Oncol. 2011;101:465–470. 21872953 10.1016/j.radonc.2011.07.019

[2] van Lin ENJ , Hoffmann AL , van Kollenburg P , Leer JW , Visser AG . Rectal wall sparing effect of three different endorectal balloons in 3D conformal and IMRT prostate radiotherapy. Int J Radiat Oncol Biol Phys. 2005;63:565–576. 16168848 10.1016/j.ijrobp.2005.05.010

[3] van Lin ENJ , Kristinsson J , Philippens MEP , et al. Reduced late rectal mucosal changes after prostate three‐dimensional conformal radiotherapy with endorectal balloon as observed in repeated endoscopy. Int J Radiat Oncol Biol Phys. 2007;67:799–811. 17161552 10.1016/j.ijrobp.2006.09.034

[4] Wortel RC , Heemsbergen WD , Smeenk RJ , et al. Local protocol variations for image guided radiation therapy in the multicenter Dutch hypofractionation (HYPRO) trial: Impact of rectal balloon and MRI delineation on anorectal dose and gastrointestinal toxicity levels. Int J Radiat Oncol Biol Phys. 2017;99:1243–1252. 28943074 10.1016/j.ijrobp.2017.07.044

[5] Vargas C , Saito AI , Hsi WC , et al. Cine‐magnetic resonance imaging assessment of intrafraction motion for prostate cancer patients supine or prone with and without a rectal balloon. Am J Clin Oncol. 2010;33:11–16. 19730351 10.1097/COC.0b013e31819fdf7c

[6] Wang KK‐H , Vapiwala N , Deville C , et al. A study to quantify the effectiveness of daily endorectal balloon for prostate intrafraction motion management. Int J Radiat Oncol Biol Phys. 2012;83:1055–1063. 22115790 10.1016/j.ijrobp.2011.07.038

[7] Smeenk RJ , Louwe RJW , Langen KM , et al. An endorectal balloon reduces intrafraction prostate motion during radiotherapy. Int J Radiat Oncol Biol Phys. 2012;83:661–669. 22099035 10.1016/j.ijrobp.2011.07.028

[8] Martin GV , Kudchadker RJ , Bruno TL , Frank SJ , Wang J . Comparison of prostate distortion by inflatable and rigid endorectal MRI coils in permanent prostate brachytherapy imaging. Brachtherapy. 2018;17:298–305. 10.1016/j.brachy.2017.09.014 29169971

[9] Osman M , Shebel H , Sankineni S , et al. Whole prostate volume and shape changes with the use of an inflatable and fexible endorectal coil. Radiol Res Pract. 2014;2014:1–6. 10.1155/2014/903747 PMC4211158 25374680

[10] Jones BL , Gan G , Diot Q , Kavanagh B . Dosimetric and deformation effects of image‐guided interventions during stereotactic body radiation therapy of the prostate using an endorectal balloon. Med Phys. 2012;39:3080–3088. 22755693 10.1118/1.4711813

[11] Jones BL , Gan G , Kavanagh B , Miften M . Effect of endorectal balloon positioning errors on target deformation and dosimetric quality during prostate SBRT. Phys Med Biol. 2013;58:7995–8006. 24169860 10.1088/0031-9155/58/22/7995

[12] Wang CW , Chong FC , Lai MK , Pu YS , Wu JK , Cheng JC . Set‐up errors due to endorectal balloon positioning in intensity modulated radiation therapy for prostate cancer. Radiother Oncol. 2007;84:177–184. 17706309 10.1016/j.radonc.2007.06.009

[13] Wang H , Dong L , O'Daniel J , et al. Validation of an accelerated demons algorithm for deformable image registration in radiation therapy. Phys Med Biol. 2005;50:2887–2905. 15930609 10.1088/0031-9155/50/12/011

[14] Thirion J‐P . Image matching as a diffusion process: an analogy with Maxwell's demons. Med Image Anal. 1998;2:243–260. 9873902 10.1016/s1361-8415(98)80022-4

[15] Van Herk M , Remeijer P , Rasch C , Lebesque JV . The probability of correct target dosage: dose‐population histograms for deriving treatment margins in radiotherapy. Int J Radiat Oncol Biol Phys. 2000;47:1121–1135. 10863086 10.1016/s0360-3016(00)00518-6

[16] Unkelbach J , Bortfeld T , Martin BC , Soukup M . Reducing the sensitivity of IMPT treatment plans to setup errors and range uncertainties via probabilistic treatment planning. Med Phys. 2009;36:149–163. 19235384 10.1118/1.3021139 PMC2673668

[17] Niemierko A . Reporting and analyzing dose distributions: a concept of equivalent uniform dose. Med Phys. 1997;24:103–110. 9029544 10.1118/1.598063

[18] Vogelius IR , Bentzen SM . Meta‐analysis of the alpha/beta ratio for prostate cancer in the presence of an overall time factor: bad news, good news, or no news? Int J Radiat Oncol Biol Phys. 2013;85:89–94. 22652106 10.1016/j.ijrobp.2012.03.004 PMC3556929

[19] Vogelius IR , Bentzen SM . Dose response and fractionation sensitivity of prostate cancer after external beam radiation therapy: a meta‐analysis of randomized trials. Int J Radiat Oncol Biol Phys. 2018;100:858–865. 29485063 10.1016/j.ijrobp.2017.12.011

[20] Suit H , Skates S , Taghian A , Okunieff P , Efird JT . Clinical implications of heterogeneity of tumor response to radiation therapy. Radiother Oncol. 1992;25:251–260. 1480770 10.1016/0167-8140(92)90244-o

[21] Niemierko A . A generalized concept of equivalent uniform dose. Med Phys. 1999;26:1101 (WE‐C2‐9 abstract).

[22] Choi B , Deasy JO . The generalized equivalent uniform dose function as a basis for intensity‐modulated treatment planning. Phys Med Biol. 2002;47:3579–3589. 12433121 10.1088/0031-9155/47/20/302

[23] Ghilezan M , Yan D , Liang J , Jaffray D , Wong J , Martinez A . Online image‐guided intensity‐modulated radiotherapy for prostate cancer: how much improvement can we expect? A theoretical assessment of clinical benefits and potential dose escalation by improving precision and accuracy of radiation delivery. Int J Radiat Oncol Biol Phys. 2004;60:1602–1610. 15590192 10.1016/j.ijrobp.2004.07.709

[24] van der Wielen GJ , Mutanga TF , Incrocci L , et al. Deformation of prostate and seminal vesicles relative to intraprostatic fiducial markers. Int J Radiat Oncol Biol Phys. 2008;72:1604–1611. 19028284 10.1016/j.ijrobp.2008.07.023

[25] Thörnqvist S , Hysing LB , Zolnay AG , et al. Treatment simulations with a statistical deformable motion model to evaluate margins for multiple targets in radiotherapy for high‐risk prostate cancer. Radiother Oncol. 2013;109:344–349. 24183863 10.1016/j.radonc.2013.09.012

[26] Meijer GJ , de Klerk J , Bzdusek K , et al. What CTV‐to‐PTV margins should be applied for prostate irradiation? Four‐dimensional quantitative assessment using model‐based deformable image registration techniques. Int J Radiat Oncol Biol Phys. 2008;72:1416–1425. 18439767 10.1016/j.ijrobp.2008.03.005

[27] Liang J , Wu Q , Yan D . The role of seminal vesicle motion in target margin assessment for online image‐guided radiotherapy for prostate cancer. Int J Radiat Oncol Biol Phys. 2009;73:935–943. 19111401 10.1016/j.ijrobp.2008.10.019 PMC2662431

[28] Deasy JO , Niemierko A , Herbert D , et al. Methodological issues in radiation dose‐volume outcome analyses: summary of a joint AAPM/NIH workshop. Med Phys. 2002;29:2109–2127. 12349932 10.1118/1.1501473

[29] Ballhausen H , Reiner M , Kantz S , Belka C , Söhn M . The random walk model of intrafraction movement. Phys Med Biol. 2013;58:2413–2427. 23503362 10.1088/0031-9155/58/7/2413

[30] Ballhausen H , Li M , Hegemann NS , Ganswindt U , Belka C . Intra‐fraction motion of the prostate is a random walk. Phys Med Biol. 2015;60:549–563. 25549204 10.1088/0031-9155/60/2/549

[31] Pommer T , Oh JH , af Rosenschöld PM , Deasy JO Simulating intrafraction prostate motion with a random walk model. Adv Radiat Oncol. 2017;2:429–436. 29114612 10.1016/j.adro.2017.03.005 PMC5605287

[32] Orlandini LC , Coppola M , Fulcheri C , Cernusco L , Wang P , Cionini L . Dose tracking assessment for image‐guided radiotherapy of the prostate bed and the impact on clinical workflow. Radiat Oncol. 2017;12:78. 28454559 10.1186/s13014-017-0815-y PMC5410096

[33] Qin A , Sun Y , Liang J , Yan D . Evaluation of online/offline image guidance/adaptation approaches for prostate cancer radiation therapy. Int J Radiat Onol Biol Phys. 2015;91:1026–1033. 10.1016/j.ijrobp.2014.12.043 25832693

[34] Wen N , Glide‐Hurst C , Nurushev T , et al. Evaluation of the deformation and corresponding dosimetric implications in prostate cancer treatment. Phys Med Biol. 2012;57:5361–5379. 22863976 10.1088/0031-9155/57/17/5361 PMC3652266

[35] Wen N , Kumarasiri A , Nurushev T , et al. An assessment of PTV margin based on actual accumulated dose for prostate cancer radiotherapy. Phys Med Biol. 2013;58:7733–7744. 24140847 10.1088/0031-9155/58/21/7733 PMC4073000

[36] Yu J , Hardcastle N , Jeong K , Bender ET , Ritter MA , Tomé WA . On voxel‐by‐voxel accumulated dose for prostate radiation therapy using deformable image registration. Technol Cancer Res Treat. 2015;14:37–47. 24354754 10.7785/tcrt.2012.500397 PMC4554757

[37] Moteabbed M , Trofimov A , Kahn FH , et al. Impact of interfractional motion on hypofractionated pencil beam scanning proton therapy and VMAT delivery for prostate cancer. Med Phys. 2018;45:4011–4019. 10.1002/mp.13091 30007067

[38] Rijkhorst EJ , Lakeman A , Nijkamp J , et al. Strategies for online organ motion correction for intensity‐modulated radiotherapy of prostate cancer: prostate, rectum, and bladder dose effects. Int J Radiat Oncol Biol Phys. 2009;75:1254–1260. 19857789 10.1016/j.ijrobp.2009.04.034

[39] Brock KK , Mutic S , McNutt TR , Li H , Kessler ML . Use of image registration and fusion algorithms and techniques in radiotherapy: report of the AAPM Radiation Therapy Committee Task Group. No. 132. Med Phys. 2017;44:e43–e76. 28376237 10.1002/mp.12256

