# Supplementary material for: Spatial and dosimetric evaluation of residual distortions of prostate and seminal vesicle bed after image‐guided definitive and postoperative radiotherapy of prostate cancer with endorectal balloon
Source: J Appl Clin Med Phys. 2020 Dec 30;22(1):226–41. doi: 10.1002/acm2.13138 (PMC7856505; doi:10.1002/acm2.13138)
Supplement: Supplementary file 1 — Fig. S1 Isotropic Hausdorff distance HDiso determined for every treatment fraction of patients treated with definitive (a) or postoperative (b) radiotherapy, respectively. Posterior Hausdorff distance HDpost per fraction for patients treated with definitive (c) or postoperative (d) radiotherapy, respectively. Fig. S2 Shift in anterior‐posterior direction assessed through the y‐component of the deformation vector at point Pworst for every treatment fraction of patients treated with definitive (a) or postoperative (b) radiotherapy, respectively. Positive values indicate a posterior shift. Relative dose Drel at point Pworst per treatment fraction for patients treated with definitive (c) or postoperative (d) radiotherapy, respectively. Fig. S3 Cumulative distribution functions of EUDSFind for 12 prostate patients. At any specified value of EUDSFind, the fraction of treatment sessions with a measured EUDSFind less than or equal to the specified value is plotted. a) Clinical plans with 6 mm margin. b) Plans with 2 mm margin. Fig. S4 Scatter plots of the median value of the distribution of the gEUDaind‐values calculated for every individual treatment fraction of each patient versus gEUDaaccum derived from the accumulated dose distributions. (a) Parameter a = −20. (b) Parameter a = −7. The plots include both 6 mm and 2 mm CTV‐to‐PTV margin plans for 12 patients. Fig. S5 Time series plots of the posterior Hausdorff distance over all treatment fractions for the four patients with worst HDpost ‐distribution (patient 7: definitive radiotherapy, patients 103, 106, and 110: postoperative radiotherapy). [file ACM2-22-226-s001.pdf]

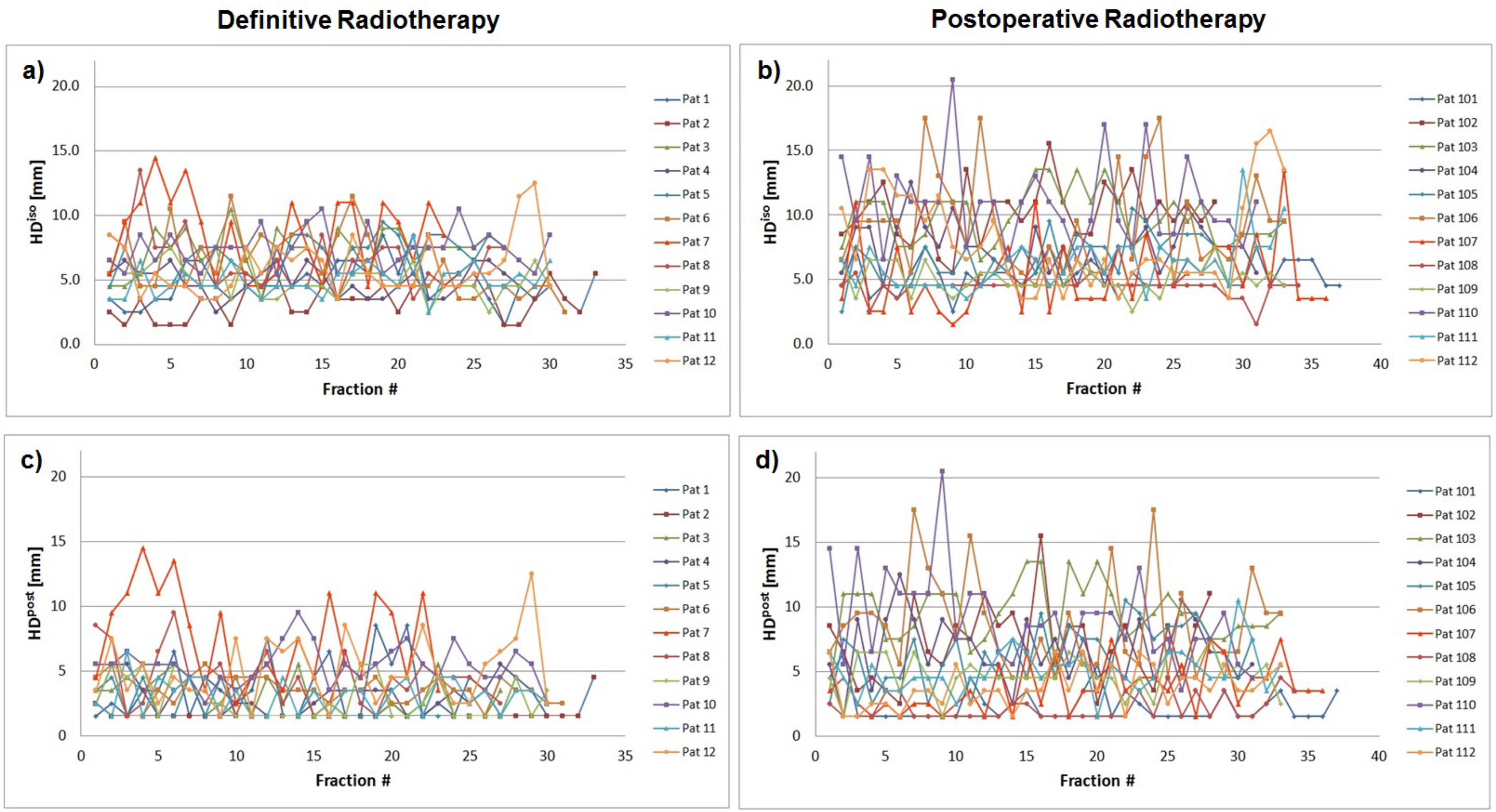

Figure S1. Isotropic Hausdorff-distance HD^{iso} determined for every treatment fraction of patients treated with definitive (a) or postoperative (b) radiotherapy, respectively. Posterior Hausdorff-distance HD^{post} per fraction for patients treated with definitive (c) or postoperative (d) radiotherapy, respectively.

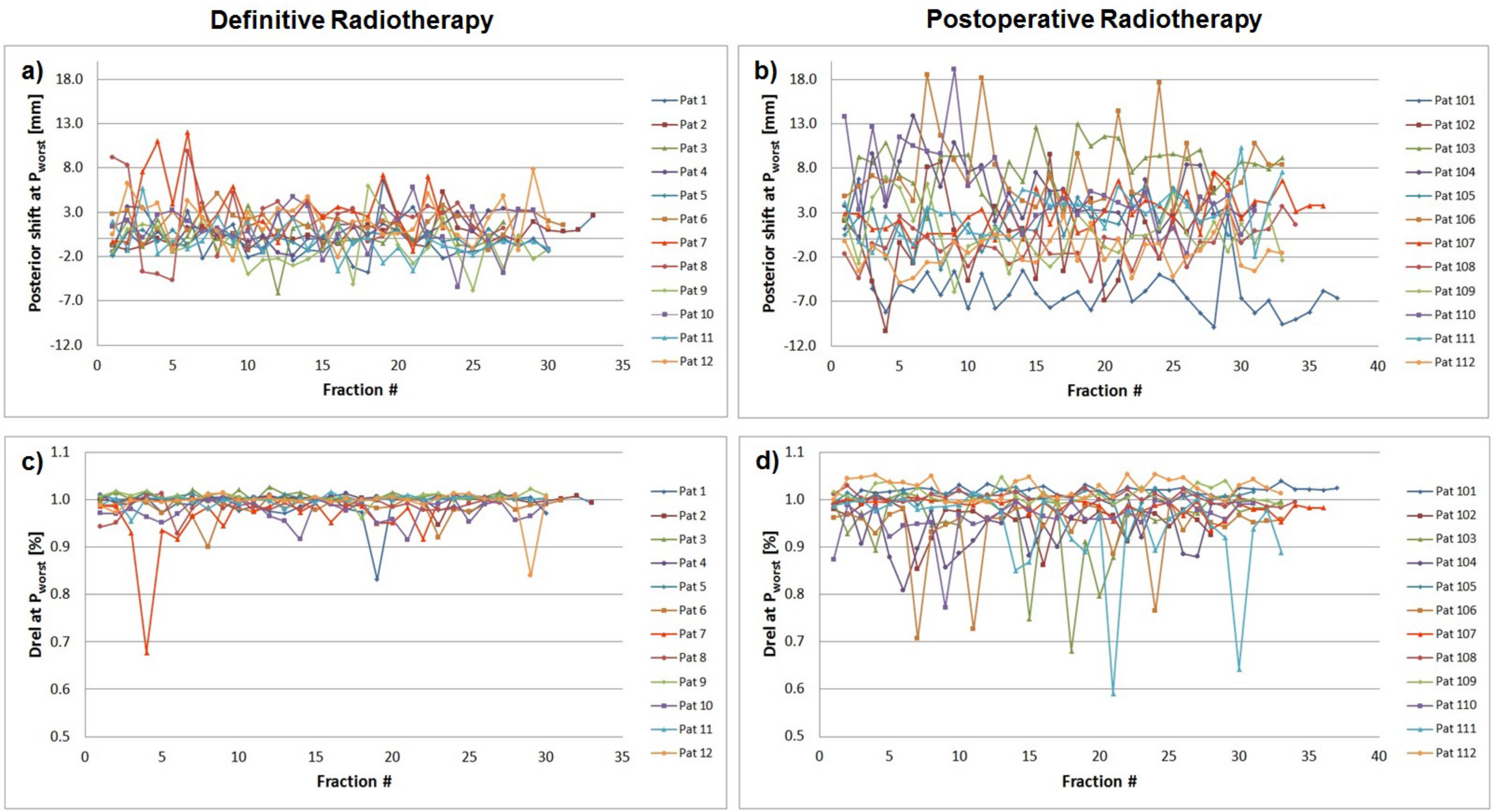

Figure S2. Shift in anterior-posterior direction assessed through the y-component of the deformation vector at point P_{worst} for every treatment fraction of patients treated with definitive (a) or postoperative (b) radiotherapy, respectively. Positive values indicate a posterior shift. Relative dose D_{rel} at point P_{worst} per treatment fraction for patients treated with definitive (c) or postoperative (d) radiotherapy, respectively.

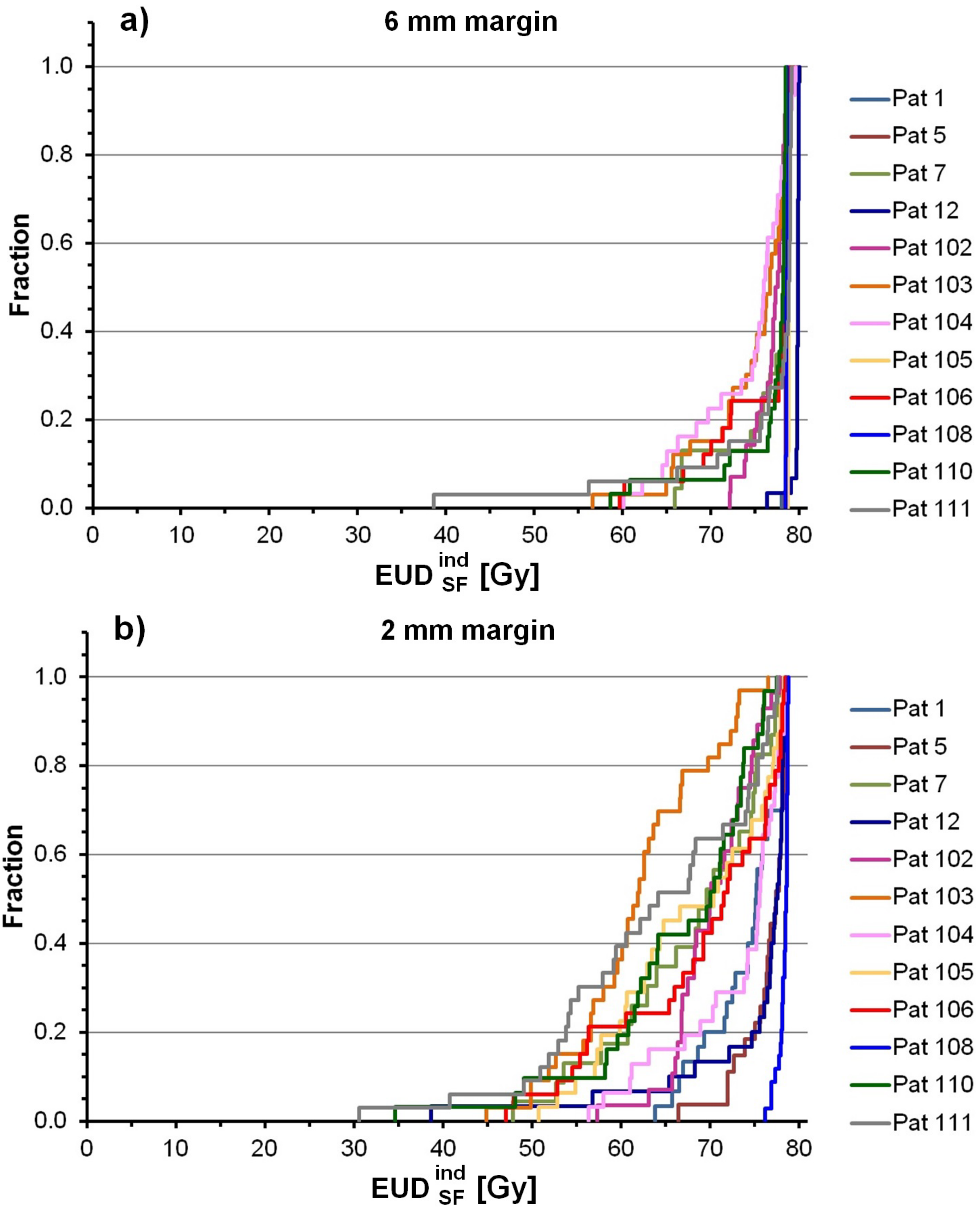

Figure S3. Cumulative distribution functions of EUD_{SF}^{ind} for 12 prostate patients. At any specified value of EUD_{SF}^{ind}, the fraction of treatment sessions with a measured EUD_{SF}^{ind} less than or equal to the specified value is plotted. a) Clinical plans with 6 mm-margin. b) Plans with 2 mm-margin.

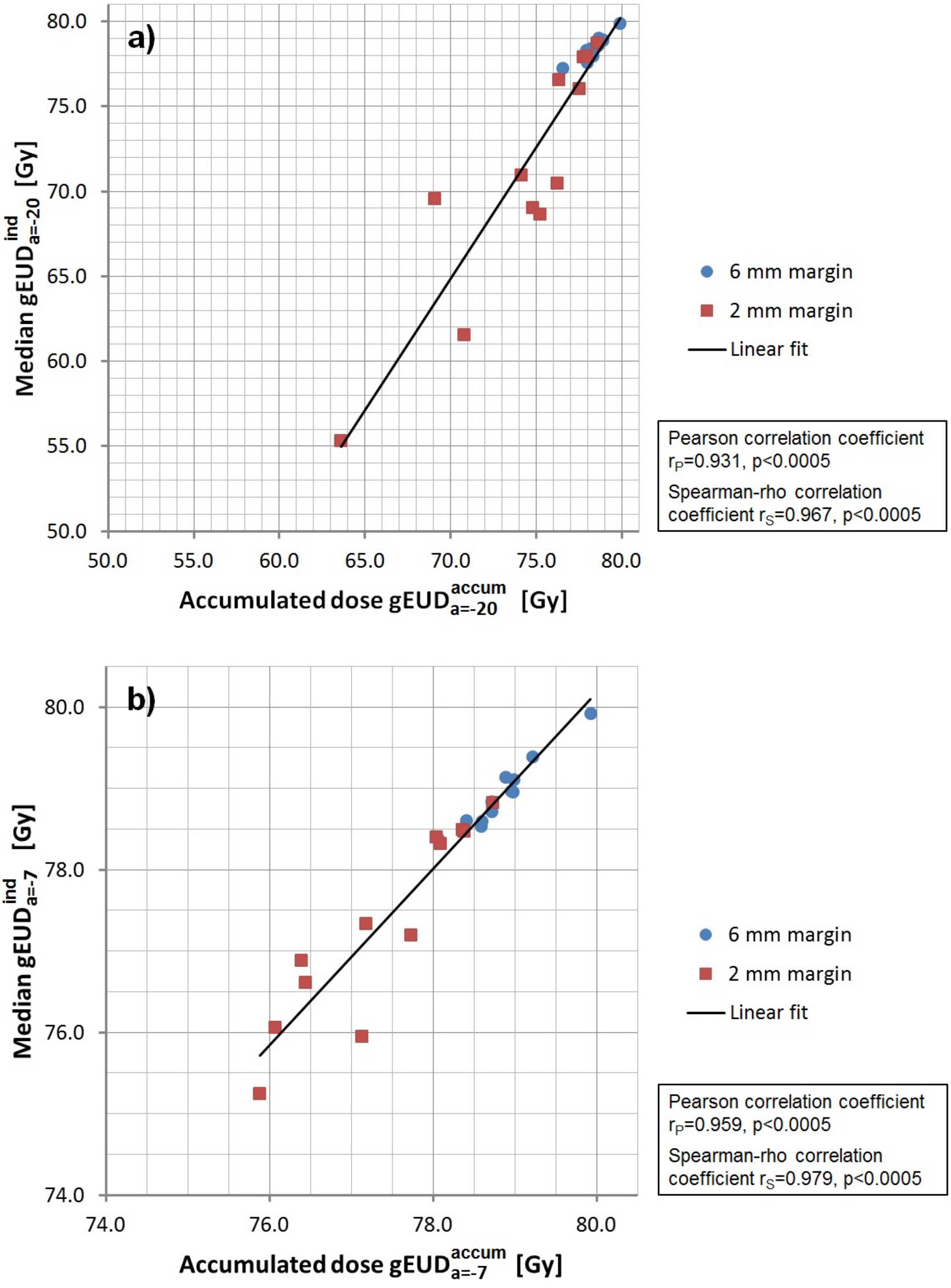

Figure S4. Scatter plots of the median value of the distribution of the $gEUD_a^{ind}$-values calculated for every individual treatment fraction of each patient versus $gEUD_a^{accum}$ derived from the accumulated dose distributions. (a) Parameter a=-20. (b) Parameter a=-7. The plots include both 6 mm and 2 mm CTV-to-PTV margin plans for 12 patients.

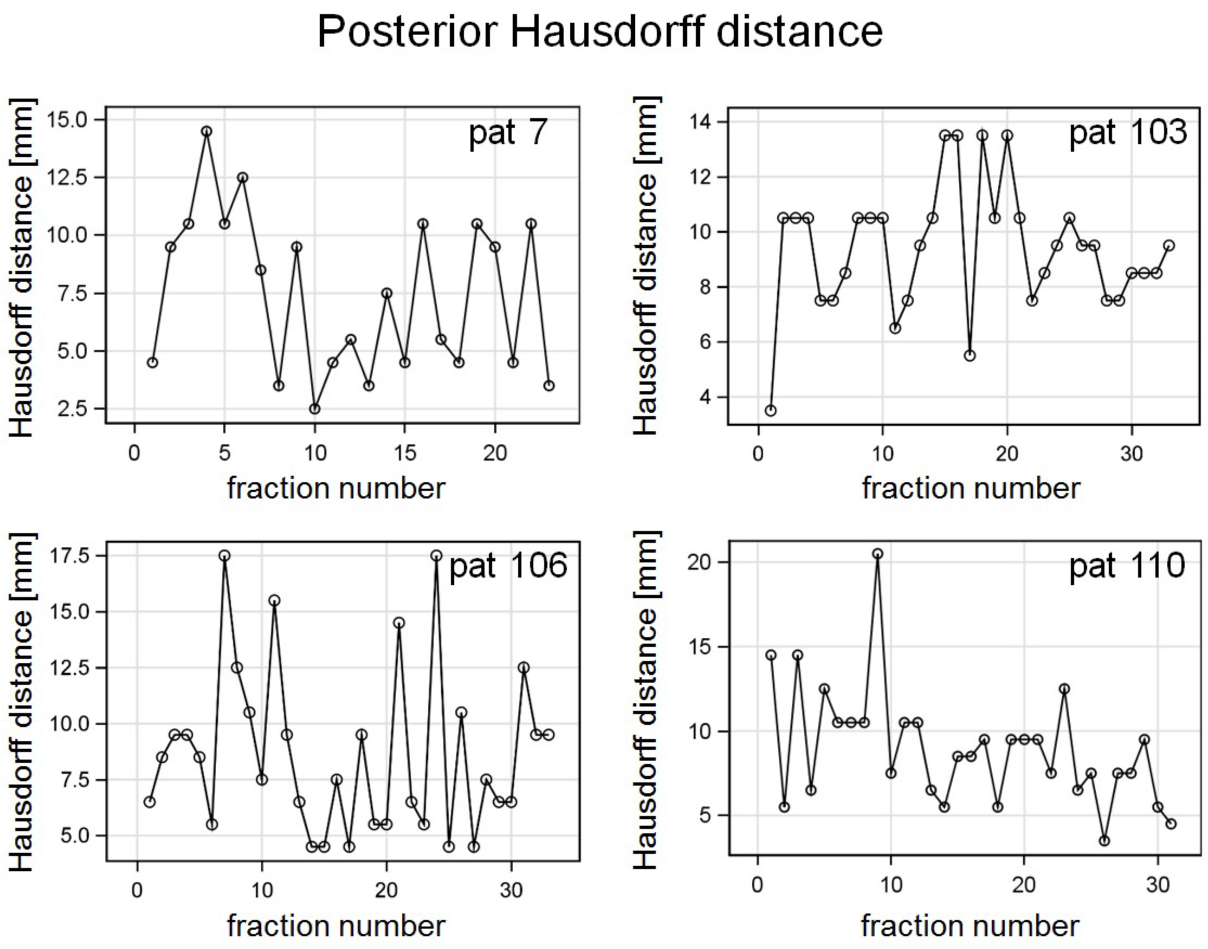

Figure S5. Time series plots of the posterior Hausdorff distance over all treatment fractions for the four patients with worst HD^{post}-distribution (patient 7: definitive radiotherapy, patients 103, 106, and 110: postoperative radiotherapy).
