# Supplementary material for: Spatial and dosimetric evaluation of residual distortions of prostate and seminal vesicle bed after image‐guided definitive and postoperative radiotherapy of prostate cancer with endorectal balloon
Source: J Appl Clin Med Phys. 2020 Dec 30;22(1):226–41. doi: 10.1002/acm2.13138 (PMC7856505; doi:10.1002/acm2.13138)
Supplement: Supplementary file 2 — Table S1 Comparison of prostate cancer patients treated with definitive and postoperative radiotherapy. Table S2 Parameters characterizing the distributions of the isotropic and posterior Hausdorff‐distances over the treatment series for prostate patients treated with definitive (patients 1‐12) or postoperative radiotherapy (patients 101‐112), respectively. Table S3 Number of fractions with selected spatial and dosimetric parameters exceeding a given threshold value. The numbers in brackets identify the corresponding patient(s). Table S4 Parameters characterizing the distributions of the deformation vector components over the treatment series for prostate patients treated with definitive (patients 1‐12) or postoperative radiotherapy (patients 101‐112). Table S5 Parameters summarizing the distributions of the anterior‐posterior deformation vector component y and of the dose deviations with respect to the treatment plan at different points of interest within the planned CTV. [file ACM2-22-226-s002.docx]

**Table S1.** Comparison of prostate cancer patients treated with definitive and postoperative radiotherapy

| Characteristic | Definitive Radiotherapy | Postoperative Radiotherapy | t-test ^b^ |
| --- | --- | --- | --- |
| CTV_plan_ [cm^3^] ^a^ | 78.5 **⎜**74.9 (41.1 – 133.1) | 104.5 **⎜**108.8 (56.8 – 142.1) | p=0.03 |
| CTV_acc_ [cm^3^] ^a^ | 127.7 **⎜**118.0 (71.7 – 183.4) | 181.7 **⎜**173.6 (100.3 – 298.3) | p=0.007 |
| PTV [cm^3^] ^a^ | 220.4 **⎜**208.1 (148.7 – 315.7) | 255.4 **⎜**261.1 (172.9 – 340.7) | p=0.1 |
| Bladder volume [cm^3^] ^a^ | 238.5 **⎜**204.9 (104.3 – 461.6) | 352.7 **⎜**326.8 (134.9 – 817.6) | p=0.1 |
| ERB volume [cm^3^] ^a^ | 62.0 **⎜**63.7 (40.2 – 74.0) | 68.7 **⎜**63.9 (42.4 – 126.1) | p=0.3 |
| ERB sagittal diameter [cm] ^a^ | 3.52 **⎜**3.57 (2.69 – 3.95) | 3.58 **⎜**3.64 (3.06 – 4.45) | p=0.7 |
| Vol(CTV_CBCTi_) outside PTV^2mm^ iso [cm^3^] ^a,c^ | 1.93 **⎜**0.65 (0 –22.6) | 4.18 **⎜**2.03 (0 – 53.5) | p<0.0005 |
| Vol(CTV_CBCTi_) outside PTV^5mm^ iso [cm^3^] ^a,c^ | 0.35 **⎜**0 (0 – 12.3) | 1.27 **⎜**0.05 (0 – 33.8) | p<0.0005 |
| Vol(CTV_CBCTi_) outside PTV^2mm^ post [cm^3^] ^a,d^ | 0.50 **⎜**0 (0 – 19.5) | 1.93 **⎜**0.43 (0 – 21.9) | p<0.0005 |
| Vol(CTV_CBCTi_) outside PTV^5mm^ post [cm^3^] ^a,d^ | 0.13 **⎜**0 (0 – 12.1) | 0.56 **⎜**0 (0 – 11.9) | p<0.0005 |

**Abbreviations:** ERB: endorectal balloon. PTV^2mm^: planned CTV (CTV_plan_) expanded isotropically by 2 mm. PTV^5mm^: CTV_plan_ expanded isotropically by 5 mm. iso: isotropic. post: posterior.

^a^ Values denote mean **⎜**median (range).

^b^ Two-sided p-value.

^c^ Volume of the daily CTV-contour (CTV_CBCTi_) outside of PTV^2mm^ (PTV^5mm^) evaluated isotropically in all directions

^d^ Volume of the daily CTV-contour (CTV_CBCTi_) outside of PTV^2mm^ (PTV^5mm^) in posterior direction

**Table S2.** Parameters characterizing the distributions of the isotropic and posterior Hausdorff-distances over the treatment series for prostate patients treated with definitive (patients 1-12) or postoperative radiotherapy (patients 101-112), respectively


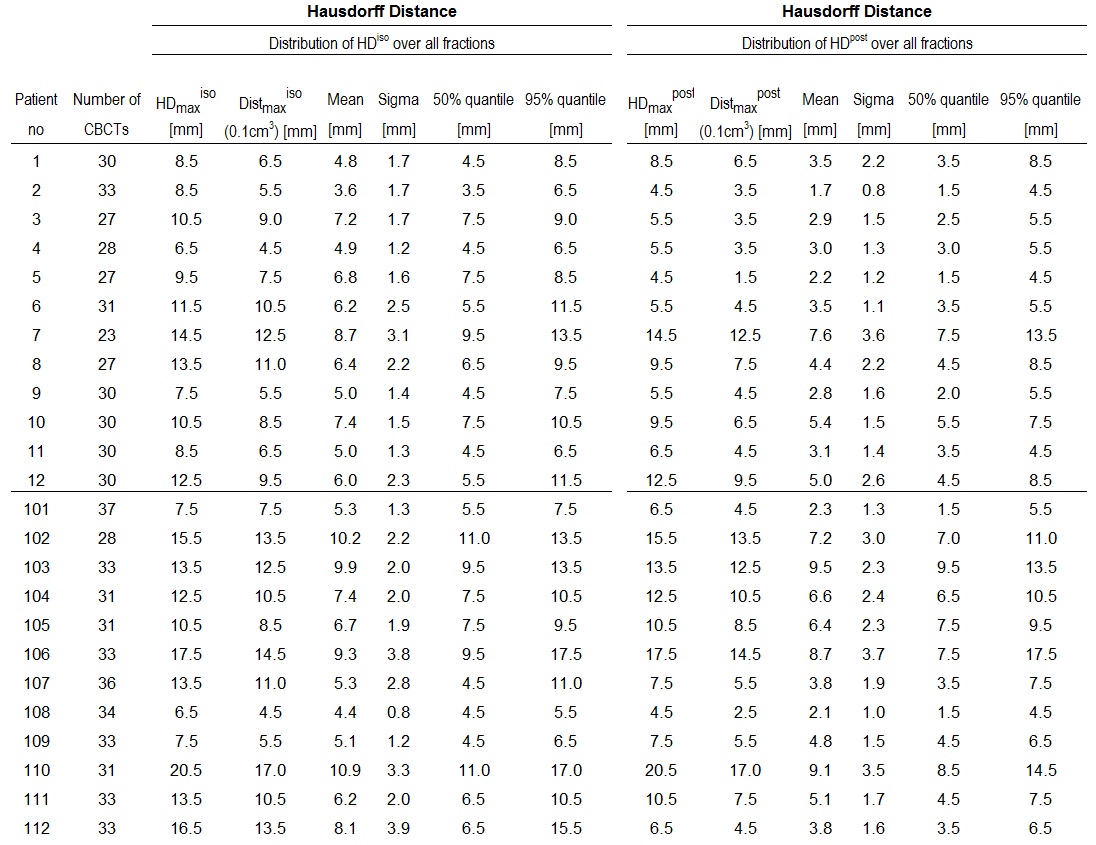


**Abbreviations:** HD_max_: maximum value of the Hausdorff-distance between the planned CTV (CTV_plan_) and the CTV_CBCTi_ in each CBCT over all treatment fractions per patient. iso: isotropic. post: posterior. Dist_max_(0.1 cm^3^): maximum value over all fractions of the isotropic margin around CTV_plan_ such that a volume of 0.1 cm^3^ of CTV_CBCTi_ is outside.

**Table S3.** Number of fractions with selected spatial and dosimetric parameters exceeding a given threshold value. The numbers in brackets identify the corresponding patient(s).

| **Threshold** | **Definitive RT** | **Postoperative RT** | **Total** |
| --- | --- | --- | --- |
| HD^iso^ > 15 mm | 0 fx | 9 fx in 4 pat  (102,106,110,112) | 9 fx in 4 pat |
| HD^iso^ > 12.5 mm | 4 fx in 3 pat  (7,8,12) | 33 fx in 8 pat  (102,103,104,106, 107,110,111,112) | 37 fx in 11 pat |
|  |  |  |  |
| HD^post^ > 15 mm | 0 fx | 5 fx in 3 pat  (102,106,110) | 5 fx in 3 pat |
| HD^post^ > 12.5 mm | 3 fx in 2 pat  (7,12) | 17 fx in 5 pat  (102,103,104,106, 110) | 20 fx in 7 pat |
|  |  |  |  |
| Posterior shift at P_worst_ > 15 mm | 0 fx | 4 fx in 1 pat  (106) | 4 fx in 2 pat |
| Posterior shift at P_worst_ > 10 mm | 2 fx in 1 pat  (7) | 22 fx in 5 pat  (103,104,106,110, 111) | 24 fx in 6 pat |
|  |  |  |  |
| Vol(CTV_CBCTi_) outside PTV^5mm^ > 10 cm^3^, isotropic | 2 fx in 1 pat  (7) | 9 fx in 3 pat  (103,106,110) | 11 fx in 4 pat |
| Vol(CTV_CBCTi_) outside PTV^5mm^ > 5 cm^3^, isotropic | 5 fx in 3 pat  (7,10,12) | 24 fx in 6 pat  (102,103,106,110, 111,112) | 29 fx in 9 pat |
|  |  |  |  |
| Vol(CTV_CBCTi_) outside PTV^5mm^ > 10 cm^3^, posterior | 2 fx in 1 pat  (7) | 1 fx in 1 pat  (110) | 3 fx in 2 pat |
| Vol(CTV_CBCTi_) outside PTV^5mm^ > 3 cm^3^, posterior | 5 fx in 1 pat  (7) | 27 fx in 5 pat  (102,103,104,106, 110) | 32 fx in 6 pat |
|  |  |  |  |
| D_rel_ at P_worst_ < 0.8 | 1 fx in 1 pat  (7) | 9 fx in 4 pat  (103,106,110,112) | 10 fx in 5 pat |
| D_rel_ at P_worst_ <0.9 | 3 fx in 3 pat  (1,7,12) | 28 fx in 6 pat  (102,103,104,106, 110,111) | 31 fx in 9 pat |

**Table S4.** Parameters characterizing the distributions of the deformation vector components over the treatment series for prostate patients treated with definitive (patients 1-12) or postoperative radiotherapy (patients 101-112)


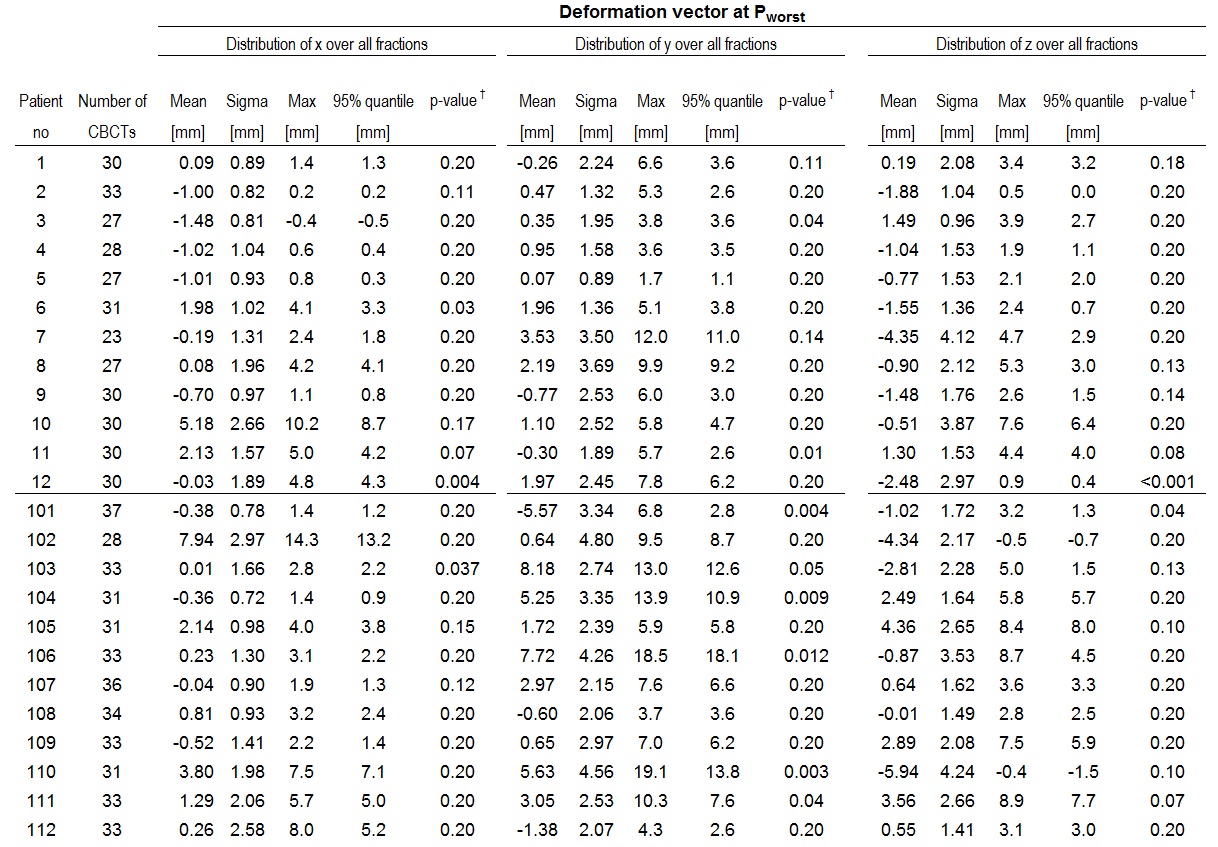


^†^ Shapiro-Wilk test for normality. Positive x-value: shift to the left; positive y-value: posterior shift; positive z-value: cranial shift.

**Table S5.** Parameters summarizing the distributions of the anterior-posterior deformation vector component y and of the dose deviations with respect to the treatment plan at different points of interest within the planned CTV

| Patient no | Point | Deformation vector component y ^a^ | | | Relative dose wrt treatment plan | | | |
| --- | --- | --- | --- | --- | --- | --- | --- | --- |
|  |  | Mean  [mm] | Sigma [mm] | Maximum [mm] | Mean  [%] | Sigma  [%] | Minimum  [%] |  |
| 7 | P_0_ | 0.7 | 3.3 | 8.8 | 100.2 | 0.5 | 99.0 |  |
|  | P_1_ | 3.2 | 3.8 | 10.7 | 97.8 | 3.3 | 88.2 |  |
|  | P_2_ | 1.3 | 3.8 | 9.7 | 99.2 | 3.8 | 85.2 |  |
|  | P_3_ | 2.2 | 3.9 | 12.1 | 96.0 | 9.8 | 66.4 |  |
|  | P_4_ | 3.1 | 3.9 | 11.6 | 98.5 | 4.2 | 86.9 |  |
|  | P_5_ | 3.8 | 3.6 | 11.2 | 95.8 | 5.8 | 75.0 |  |
|  | P_6_ | 2.5 | 3.5 | 8.6 | 99.2 | 1.1 | 96.5 |  |
|  | P_worst_ | 3.5 | 3.5 | 12.0 | 95.6 | 6.6 | 67.8 |  |
|  | all points | 2.5 | 3.7 | 12.1 | 97.8 | 5.4 | 66.4 |  |
| 103 | P_0_ | 0.1 | 2.1 | 2.9 | 99.5 | 0.5 | 98.6 |  |
|  | P_1_ | 1.2 | 2.0 | 5.5 | 100.1 | 0.4 | 99.1 |  |
|  | P_2_ | 0.5 | 2.4 | 5.2 | 98.9 | 1.1 | 95.0 |  |
|  | P_3_ | -2.3 | 2.4 | 3.5 | 100.2 | 1.2 | 98.4 |  |
|  | P_4_ | 5.1 | 2.7 | 10.3 | 99.6 | 1.2 | 94.4 |  |
|  | P_worst_ | 8.2 | 2.7 | 13.0 | 95.3 | 7.7 | 68.1 |  |
|  | all points | 2.1 | 4.2 | 13.0 | 98.9 | 3.6 | 68.1 |  |
| 106 | P_0_ | -0.7 | 3.0 | 3.4 | 102.5 | 1.6 | 97.7 |  |
|  | P_1_ | 1.7 | 2.8 | 6.1 | 99.1 | 2.1 | 91.3 |  |
|  | P_2_ | 0.7 | 3.3 | 6.4 | 98.5 | 3.0 | 85.6 |  |
|  | P_3_ | 1.7 | 3.1 | 10.3 | 99.0 | 3.9 | 83.0 |  |
|  | P_4_ | 0.8 | 3.4 | 8.1 | 99.9 | 1.1 | 95.1 |  |
|  | P_worst_ | 7.7 | 4.3 | 18.5 | 94.0 | 7.0 | 70.7 |  |
|  | all points | 2.0 | 4.3 | 18.5 | 98.8 | 4.4 | 70.7 |  |
| 110 | P_0_ | -4.3 | 4.8 | 3.3 | 100.6 | 0.9 | 98.7 |  |
|  | P_1_ | -0.3 | 3.0 | 5.7 | 100.0 | 0.7 | 98.3 |  |
|  | P_2_ | 0.5 | 3.0 | 7.4 | 98.6 | 1.1 | 94.9 |  |
|  | P_3_ | 1.9 | 2.9 | 10.3 | 98.9 | 0.8 | 97.2 |  |
|  | P_4_ | -4.4 | 4.8 | 2.7 | 98.5 | 6.0 | 67.0 |  |
|  | P_worst_ | 5.6 | 4.6 | 19.1 | 96.1 | 4.4 | 77.3 |  |
|  | all points | -0.2 | 5.2 | 19.1 | 98.8 | 3.4 | 67.0 |  |

^a^ Positive y-value: shift in posterior direction
